# Molecular subtypes and differentiation programmes of glioma stem cells as determinants of extracellular vesicle profiles and endothelial cell-stimulating activities

**DOI:** 10.1080/20013078.2018.1490144

**Published:** 2018-07-17

**Authors:** C. Spinelli, L. Montermini, B. Meehan, A. R. Brisson, S. Tan, D. Choi, I. Nakano, J. Rak

**Affiliations:** a Department of Pediatrics, McGill University, The Research Institute of the McGill University Health Centre, Montreal, Canada; b UMR-CBMN CNRS, University of Bordeaux, IPB, France; c Department of Neurosurgery, University of Alabama at Birmingham, Birmingham, AL, USA

**Keywords:** Glioblastoma, glioma stem cells, extracellular vesicles, proteome, angiogenesis

## Abstract

We have previously uncovered the impact of oncogenic and differentiation processes on extracellular vesicles (EVs) in cancer. This is of interested in the context of glioma stem cells (GSC) that are responsible for recurrent nature of glioblastoma multiforme (GBM), while retaining the potential to undergo differentiation and self renewal.  GSCs reside in vascular niches where they interact with endothelial cells through a number of mediators including bioactive cargo of EVs. GSCs can be classified as proneural (PN) or mesenchymal (MES) subtypes on the basis of their gene expression profiles and distinct biological characteristics. In the present study we investigated how GSC diversity and differentiation programmes influence their EV-mediated communication potentials. Indeed, molecular subtypes of GBMs and GSCs differ with respect to their expression of EV-related genes (vesiculome) and GSCs with PN or MES phenotypes produce EVs with markedly different characteristics, marker profiles, proteomes and endothelial stimulating activities. For example, while EVs of PN GSC are largely devoid of exosomal markers their counterparts from MES GSCs express ample CD9, CD63 and CD81 tetraspanins. In both GSC subtypes serum-induced differentiation results in profound, but distinct changes of cellular phenotypes including the enhanced EV production, reconfiguration of their proteomes and the related functional pathways. Notably, the EV uptake was a function of both subtype and differentiation state of donor cells. Thus, while, EVs produced by differentiated MES GSCs were internalized less efficiently than those from undifferentiated cells they exhibited an increased stimulatory potential for human brain endothelial cells. Such stimulating activity was also observed for EVs derived from differentiated PN GSCs, despite their even weaker uptake by endothelial cells. These findings suggest that the role of EVs as biological mediators and biomarkers in GBM may depend on the molecular subtype and functional state of donor cancer cells, including cancer stem cells.

**Abbreviations**: CryoTEM: cryo-transmission electron microscopy; DIFF: differentiated GSCs; EGF: epidermal growth factor; DUC: differential ultracentrifugation; EV: extracellular vesicle; FGF: fibroblast growth factor; GBM: glioblastoma multiforme; GFAP: glial fibrillary acidic protein; GO: gene ontology; GSC: glioma stem cells; HBEC-5i: human brain endothelial cells; MES: mesenchymal cells; MTS - [3-(4,5-dimethylthiazol-2-yl)-5-(3-carboxymethoxyphenyl)-2-(4-sulfophenyl)-2H-tetrazolium, inner salt; PMT1: proneural-to-mesenchyman transition cell line 1; PN: proneural cells; TEM: transmission electron microscopy; WB: western blotting

## Introduction

Glioblastoma multiforme (GBM) is the most frequent and lethal form of primary astrocytic brain tumour. In spite of decades of intense research, GBM continues to be associated with extremely poor outcomes, with a median survival after diagnosis of less than 15 months [1]. The main reason for this dismal course is the high rate of tumour recurrence, which is associated with both the extremely infiltrative nature of glioblastoma growth and a frequently occurring resistance to conventional treatments, such as chemoradiation therapy [2].

While GBMs share certain pathognomonic histological hallmarks, such as high vascularity, pseudopalisading necrosis and infiltrative growth patterns, these tumours are otherwise highly heterogeneous at the molecular and cellular levels [2,3]. This diversity is underscored by a broad spectrum of recurrent oncogenic driver mutations, including amplification of the epidermal growth factor receptor (EGFR), EGFR variant III mutation (EGFRvIII), isocitrate dehydrogenase 1 R132C (IDH1 R132C) mutation and multiple other changes [4]. Molecularly, GBMs converge on a handful of distinct molecular groupings, of which The Cancer Genome Atlas (TCGA) identified four gene expression subtypes designated as: proneural (PN), neural (NEU), classical (CL) and mesenchymal (MES), which are regarded as biologically different diseases [5]. Superimposed on this complexity is also the evidence for intra-tumoural diversity of GBM cells [6], their rapid genetic evolution [7] and a frequently observed shift of PN GBM towards MES-like phenotype in the course of therapy [8].

At the functional level, the evolving GBM cell populations are defined by the properties of glioma stem cells (GSCs) and their progeny [9–11]. GSCs are thought to carry tumour initiation potential and play essential roles in tumour propagation, repopulation, therapeutic resistance and relapse. While capable of self-renewal, these cells retain the ability to activate cellular programmes that result in generation of rapidly dividing, committed and differentiated bulk GBM cell populations [11,12]. GSC maintenance and fate rely upon external regulatory interactions with the brain microenvironment, of which vascular niche is thought to play a critical role [13]. Indeed, both the proximity to capillaries and pro-angiogenic activity of GSCs suggest the existence of crucial communication pathways between these cells and the endothelium [14,15].

Recent studies revealed that GSCs are heterogeneous and exist as at least two phenotypic variants, which, on the basis of their gene expression profiles and cellular properties, have been designated as either PN or MES GSC subtypes [16,17]. This diversity raises new questions as to whether distinct GSC populations and their progenies employ similar or different mechanisms of interactions with the vascular system and what are the related pathways and mediators of this communication.

Intercellular communication in GBM is of considerable interest as an implicit ramification of the cellular complexity in this disease and due to the emerging experimental evidence [18–21]. In this regard, a unique role is attributed to extracellular vesicles (EVs) known to emanate from GBM cells and GSCs and endowed with the ability to carry and transmit regulatory signals to recipient cells including angiogenic endothelium, thereby eliciting a range of biological responses [18,22]. EVs are spherical membrane structures that cells emit in a regulated manner from either their surfaces (ectosomes) or from within endosomal compartments (exosomes). Other processes of EV formation include amoeboid phenotype, specialized membrane structures, such as blebs or cilia, and cell disintegration following apoptotic death [23]. Depending on their origin and biogenesis EVs differ in size, density and pre-assembled molecular content (cargo), which includes bioactive proteins, lipids and nucleic acids (RNA, DNA). In some instances, distinct biological activities could be assigned to separable EV subsets. For example, exosome-like EVs are usually below 150 nm in diameter, rich in tetraspanins (CD9, CD63, CD81), carry markers of multivesicular bodies (MVBs) [24] and can be sedimented at high centrifugal forces of 1 × 10^5^ × g (100K). Larger EVs, such as ectosomes, or microvesicles may sediment at lower speeds (10K) and possess different biological properties [25].

The release and biological effects of EVs in GBM are increasingly well characterized [26], including the contribution of oncogenic pathways [18,27,28]. There is also emerging evidence for the role of EVs in GBM-associated angiogenesis [18,22,29,30]. In this regard, we proposed earlier that molecular diversity of GBM subtypes would likely translate into a corresponding diversity of EV biogenesis pathways, cargo assembly mechanisms and biological activity [17]. However, this EV heterogeneity is poorly described and little is known about its implications for angiogenesis-related EV characteristics. Here, we showed that PN and MES GSC lines produce vastly different populations of EVs, including varying EV profiles, marker distributions and proteomes. Moreover, serum-induced differentiation of these GSC subsets results in considerable differences in cellular phenotype, increased EV emission, and altered molecular content. Notably, differentiation of MES GSCs leads to production of small EVs with heightened ability to stimulate growth of brain endothelial cells. These results point to a role of GSC heterogeneity and differentiation potential in EV-mediated communication of GBM cells with the vascular system.

## Material & Methods

### Cell lines

GSC lines were isolated as sphere forming units from surgical samples of GBM in the laboratory of Ichiro Nakano (IN). Two of these cell lines, designated as 157 and 83, were selected as representative of PN and MES subtype, respectively. All GSCs were maintained as sphere cultures unless otherwise indicated, in DMEM-F12 media (GIBCO # 11320033) supplemented with EGF (GIBCO # PHG0311L), FGF (GIBCO # PHG0261), Heparin 0.2% (STEMCELL # 07980), B27 serum free supplement (GIBCO #17504044), Glutamax (GIBCO # 35050061) and 1% penicillin-streptomycin (P/S) (GIBCO # 15070063). In experiments involving GSC differentiation the cells were maintained in DMEM-F12 supplemented with 10% FBS, 1% P/S and 1% Glutamax. An immortalized human brain endothelial cell line (HBEC-5i) was purchased from the American Type Culture Collection (ATCC; Manassas, VA) and the cells were grown in DMEM-F12 media supplemented with 10% FBS, 1% P/S and 40 µg/mL endothelial growth supplement (ECGS) (Sigma E2759).

### EV isolation and fractionation

EVs were purified by differential centrifugation (Beckman TLA100.2 rotor) from the indicated conditioned medium of monolayer cell cultures [31]. GSC were grown in their media, while differentiated cells were maintained in EV-depleted FBS. After three days, cell debris was eliminated by centrifugation at 2,000 × g for 20 min. The supernatant was then concentrated (centrifuged at 3,500 × g for 20 min) using Amicon Ultra-15 Centrifugal Filter Units −100KDa- (Millipore # UFC905008) to a final volume of 1mL. Concentrated conditioned medium was then centrifuged at 10,000 × g for 45 min to precipitate the 10K pellet (ectosome/microvesicle-like EVs). For isolation of exosome-like EVs, the supernatant remaining after the first centrifugation was passed through 0.22 μm filter and then centrifuged at 110,000 × g for 70 min. The resulting EV pellet was re-suspended in filtered 1× PBS or RIPA buffer and stored at −80°C (designated for simplicity as 100K).

### Gene expression profiling by RT-qPCR

Total RNA was extracted from the cells using TRIzol Reagent (Invitrogen # 15596026) and RNeasy Mini Kit (Qiagen # 74104, Mississauga, ON, Canada) according to the manufacturer’s recommendations. Starting from 0.5 μg of RNA, cDNA was synthetized using RT2 First Strand Kit (Qiagen # 330404). We designed a custom-made RT2 Profiler PCR Array with validated primers directed at 87 genes implicated in EV biogenesis and activity (Table S1). In combination with RT2 SYBR Green Mastermix (Qiagen # 330503), the expression profiles of these genes were quantified simultaneously using RT-qPCR machine (Roche Light Cycler 96) following the manufacturer’s recommendations. The results were analysed using Excel and a heat map was created using MeV software. Quantitative PCR of GFAP mRNA was performed in a total reaction volume of 25 μl in 96-well reaction plates using One Step RT-PCR kit (Qiagen # 210210) and 250 nM of the following primers: GFAP (Forward 5ʹ-cgatcaactcaccgccaaca-3ʹ Reverse 5ʹ-gggtgagtttcttgttagttgg-3ʹ), GAPDH (Forward 5ʹcgatcaactcacggccaaca-3ʹ Reverse 5ʹgagtcaacggatttggtcgt-3ʹ). The amplification conditions for the Light Cycler 480 SW 1.5.1 (Roche) consisted of an initial step of 5 min at 95°C for enzyme activation followed by 40 cycles of 15 sec at 95°C and 1 min at 60°C. The delta–delta CT method was used as described by Perkin-Elmer Applied Biosystems to determine the relative levels of mRNA expression between undifferentiated (GSC) and differentiated (DIFF) GSCs.

### Nanoparticle tracking analysis

The number and size of emitted EVs were analysed using NS500 nanoparticle tracking analysis system (NTA; Nanosight, Amesbury, UK). Culture media were centrifuged at 400 g for 10 min to remove cells, and 1:300 aliquots were loaded into the flow chamber. Similar analysis was also carried out on the 100K fraction of conditioned medium ultracentrifugate. At least 3 recordings, of 30 s each, were obtained under automatic detection and batch processing settings.

### Protein quantification and western blot (WB)

RIPA buffer containing protease inhibitor (Roche, Mississauga, ON, Canada) was used to isolate total proteins from cells or EVs. Lysates were incubated on ice for 5 min, centrifuged at 13,000 rpm for 10 min at 4°C, and solubilized proteins were quantified using the Pierce Micro BCA^TM^ Protein Assay (Thermo Scientific, Rockford, IL, USA). Proteins were resolved using sodium dodecyl sulphate–polyacrylamide gel electrophoresis (SDS-PAGE), at 10%, and transferred to polyvinylidene difluoride membranes (PVDF; Biorad, Mississauga, ON, Canada). The membranes were probed with indicated primary antibodies, and appropriate horseradish peroxidise (HRP)-conjugated secondary anti-mouse (Biorad # 170–6516), or anti-rabbit (Cell Signaling # 7074S) antibodies. Chemiluminescence (GE Healthcare) was visualized using ChemiDoc MP system (Biorad). Primary antibodies included: rabbit anti-CD81 (ab155760 Abcam), rabbit anti-CD9 (ab92726 Abcam), rabbit anti-CD63 (ab134045 Abcam or 556019 BD), rabbit anti-CD82 (12439 Cell signalling), rabbit anti-ANXA6 (ab31026 Abcam), rabbit anti-CAV1 (ab2910 Abcam), rabbit anti-SYN (ab133267 Abcam), mouse anti-FLOT1 (610821 BD), rabbit anti-RAB6B (ab206110 Abcam), mouse anti-RAB27A (5873-MO2 Abnova), rabbit anti-WNT11 (ab31962 Abcam), rabbit anti-WNT16 (ab109437 Abcam) and rabbit anti-ITGα5 (ab117611 Abcam).

### Proteomic and gene ontology (GO) analysis

Isolated proteins from exosome-like EVs were loaded onto an 10% SDS-PAGE pre-cast gel (BioRad) and run into the stacking gel to remove any detergents and salts. The bands were cut, followed by reduction with DTT, alkylation with iodoacetic acid, and digested with trypsin [32]. The lyophilized peptides were re-solubilized in 0.1% aqueous formic acid 2% acetonitrile, the peptides were loaded onto a Thermo Acclaim Pepmap (Thermo, 75 μM ID × 2 cm C18 3 μM beads) pre-column and then onto an Acclaim Pepmap Easyspray (Thermo, 75 μM × 15 cm with 2 μM C18 beads) analytical column separation using a Dionex Ultimate 3000 uHPLC at 220 nl/min with a gradient of 2–35% organic (0.1% formic acid in acetonitrile) over 2 h. Peptides were analysed using a Thermo Orbitrap Fusion mass spectrometer operating at 120,000 resolution (FWHM in MS1, 15,000 for MS/MS) with HCD sequencing all peptides with a charge of 2+ or greater. The raw data were converted into *.mgf format (Mascot generic format) searched using Mascot 2.3 against human sequences (Swissprot). The database search results were loaded onto Scaffold Q+ Scaffold_4.4.8 (Proteome Sciences) for spectral counting, statistical treatment and data visualization. We also performed a functional enrichment analysis using FunRich21, an open-source software developed for the analysis of data from EV-specialized databases including Vesiclepedia and Exocarta. The open-source platform GeneMania Cytoscape was used for representation of changes in functional clusters of EV-associated proteins released from mesenchymal and proneural GSCs in their undifferentiated and differentiated states.

### EVs labelling and internalization assays

EVs isolated from cells were labelled with PKH26 (#MINI26, Sigma-Aldrich) red fluorescent dye as described earlier [33,34]. Briefly, EVs were re-suspended in 250 μL of 2× Diluent C. The 2 × Dye Solution (4 × 10^–6^ M) in Diluent C is prepared by adding 1 μL of the PKH26 ethanolic dye solution (# P9691) to 250 mL of Diluent C in a polypropylene centrifuge tube and mixed well to disperse. We then rapidly added the 250 μL of 2× EV suspension to 250 μL of 2× Dye Solution and immediately mixed the sample by pipetting. The EVs/dye suspension was incubated for 5 min with periodic mixing and the staining was stopped by adding an equal volume of FBS for 1 min and then an equal volume of complete medium. The labelled EVs were washed twice with PBS, re-suspended in PBS  (by resuspension-ultracentrifugation) and incubated overnight with 1.5 × 10^5^ recipient cells (HBEC-5i cells), which were then analysed by FACS for the PKH26 fluorescence transfer.

### Cell growth/survival assays (MTS assay)

Cell titre 96 (Promega # 43580) was used to measure *in vitro* cell growth/viability in the presence of EV treatments. As indicated, 7 × 10^3^ HBEC-5i cells/well were seeded in 96 well plates in full media for 24 h. The following day, the cells were washed and treated with 30 μg (protein)/mL of EV preparations in DMEM containing 1% FBS. The absorbance at 490 nm was read at time intervals indicated and the signal reflective of viable cell numbers was assessed for up to 6 days.

### Transmission Electron Microscopy (TEM) and Cryo-TEM

Cells were processed for ultramicrotomy as follows. The cells were centrifuged at 5,000 rpm to yield a pellet, which was re-suspended in 0.1 M sodium cacodylate buffer (pH 7.4), fixed in 2.5% glutaraldehyde, post-fixed with 1% osmium and embedded in Epon resin after acetone dehydration. Thin sections (100 nm) were stained successively with 4% uranyl acetate and Reynold's lead 5%. EVs were washed once by resuspension-unltracentrifugation using 0.1 M sodium cacodylate buffer (pH 7.4) and fixed with 2.5% glutaraldehyde in the same buffer. TEM observation of cells and EVs was performed with a FEI Tecnai 12 BioTwin 120 kV TEM with a AMT XR80C CCD Camera System. For immuno-cryo-TEM, 10-nm gold nanoparticles (NPs) were conjugated with anti-CD63 mAbs following procedures previously described by Arraud et al [35]. Fixed EV pellets were diluted 10× with a buffer containing 150 mM NaCl, 2 mM CaCl2 and 10 mM HEPES, pH 7.4, and labelled for 1 h with 1–4 × 10^15^ anti-CD63-mAb-gold-NP/L. Immuno-gold labelled samples were processed for cryo-TEM as follows. A 4-μL aliquot was deposited on an EM grid coated with a perforated carbon film; the liquid was blotted with a filter paper and the grid was quickly plunged into liquid ethane using a Leica EMCPC cryo-chamber. EM grids were stored under liquid nitrogen prior to EM observation. Cryo-TEM was performed with a Tecnai F20 (FEI, USA) microscope equipped with a USC1000-SSCCD camera (Gatan, USA).

### Data analysis

All experiments were reproduced at least three times with similar results unless otherwise indicated. The numerical values were presented as mean ± SD, and statistical analysis was performed using t test, at the threshold p value of 0.05.

## Results

### The expression of vesiculation-related genes reflects molecular subtypes of human GBM

We reasoned that the molecular heterogeneity of GBMs not only reflects the intracellular driver events but may also impinge upon pathways of intercellular communication. Since EV biogenesis, release and cargo are regulated by oncogenic pathways, which also define molecular subtypes of GBM, we surmised that genes involved in cellular vesiculation (vesiculome) would be expected to be expressed in a non-random manner, with a degree of subtype specificity [17]. To explore this notion in more detail, we performed an extended *in silico* analysis of 87 vesiculation-related transcripts (Table S1) included in the TCGA gene expression data set, in which samples of newly diagnosed GBM were annotated for classifiers of PN, NEU, CL and MES subtypes [5]. Indeed, we observed that several vesiculation-related genes exhibited subtype-specific expression patterns (Figure S1). For example, MES GBMs (red arrows) were enriched for CAV1, CD44, CD63, RAB27A, SDCBP and SMPDL3A, while PN (blue arrows) tumours contained higher levels of transcripts for ANXA6, CD81, hnRNPA2B1, YBX1 (RNA binding proteins) and RAB35 (Figures S1 and S2). While these profiles are intriguing and suggestive of differential roles of EVs in the biology of GBM subtypes, they include signals from multiple cellular sources and stromal cell populations making more mechanistic linkages difficult to discern.

### Differential expression of vesiculation-related genes by molecular subtypes of GSCs

**Figure 1.**
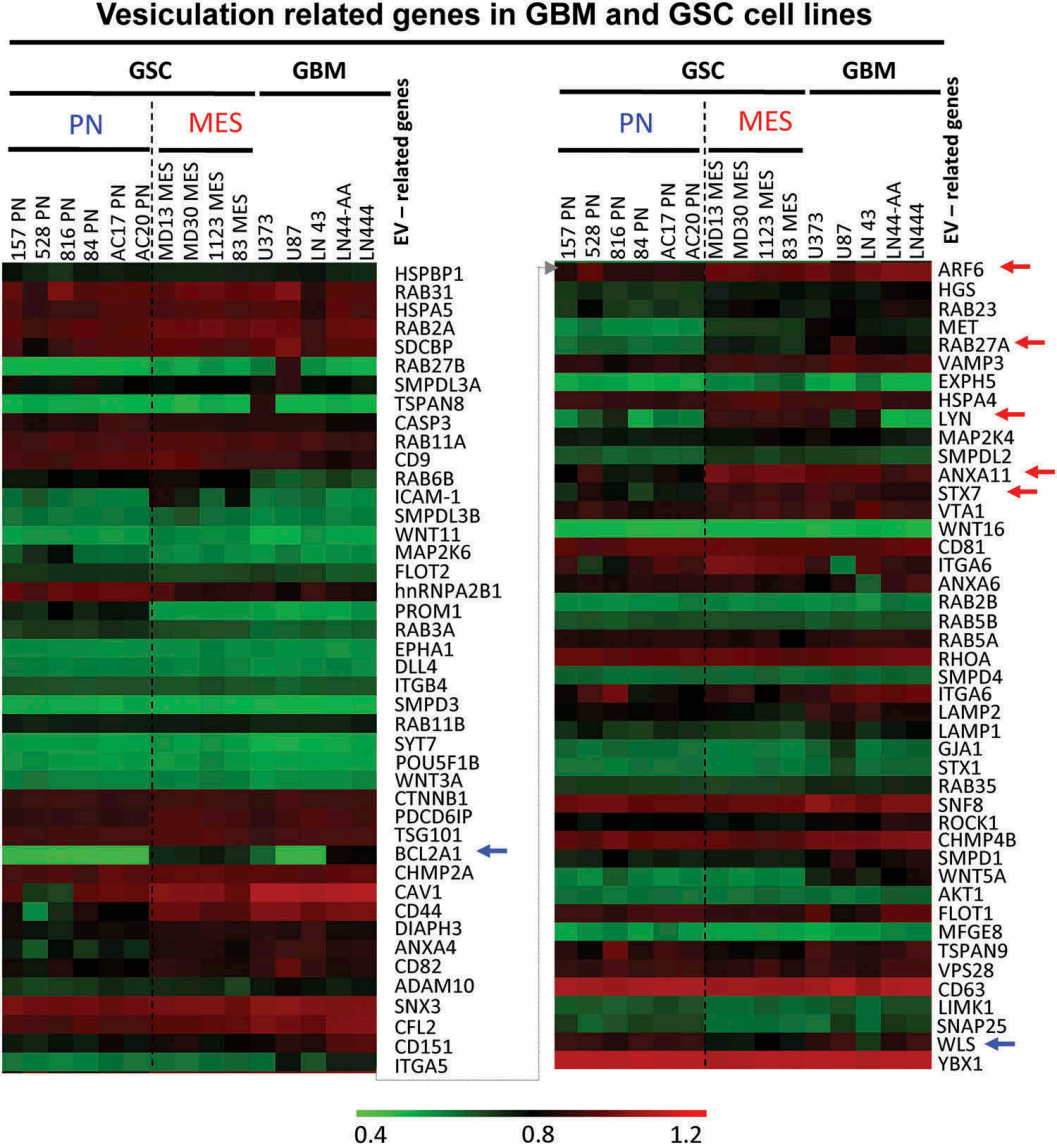
Expression of vesiculation-related genes in datasets of glioma cell lines and stem cell lines. Representation of 90 genes of which 87 are known to regulate EV biogenesis, or contribute to EV cargo is shown in Table S1. The remaining genes are elements of the GBM landscape. This information was extracted from transcriptomes of 15 glioma cell lines, including 10 glioma stem cell isolates (GSC) (6 PN, 4 MES) and 5 established GBM cell lines (GBM). The hierarchical clustering shows differential expression of genes based on stem cells subtypes. Blue arrows represent upregulated genes in PN GSC, while red arrows represent upregulated genes in MES GSC.

The recent evidence for molecular and functional heterogeneity among GSCs, and the existence of their PN and MES subtypes raises the possibility of a corresponding diversity among EV-mediated cell-interactive networks [17,36]. To glean some related insights from the existing GSC gene expression datasets (GEO GSE67089), we extracted mRNA levels of 90 genes including known EV regulators, markers, effectors and selected cargo proteins and we clustered them according to their PN or MES GSC signatures. Interestingly, such supervised clustering analysis (Figure 1) shows marked differences between the expression profiles of EV-related transcripts in a series of PN and MES GSC lines. Thus, several such genes were preferentially upregulated in MES GSCs, including ARF6, RAB27A, ANXA11, STX7 relative to PN GSCs (Figure 1 – red arrows). While PN GSCs exhibited predictably higher levels of lineage-related transcription factors (SOX2, OLIG2), their expression of common EV regulators was often relatively low, as exemplified by RAB27A and ARF6, with notable changes (relative to MES GSC) in genes involved in intracellular vesicular transport (STX7), WNT signalling (WLS) and regulation of cell survival (BCL2A1). Interestingly, while PN and MES GSC subsets were so classified due to their transcriptional profiles analogous to those of PN and MES GBMs, several elements of the GSC vesiculome are not identical to those found in the corresponding GBM subtypes, or to the established non-stem GBM cell lines (right section of the heat map in Figure 1; Figure S1-2). This suggests that GSC stemness may influence the expression of genes related to EV biogenesis, emission, and cargo, which may lead to distinct biological properties of GSC EVs and have functional and translational significance.

**Figure 2.**
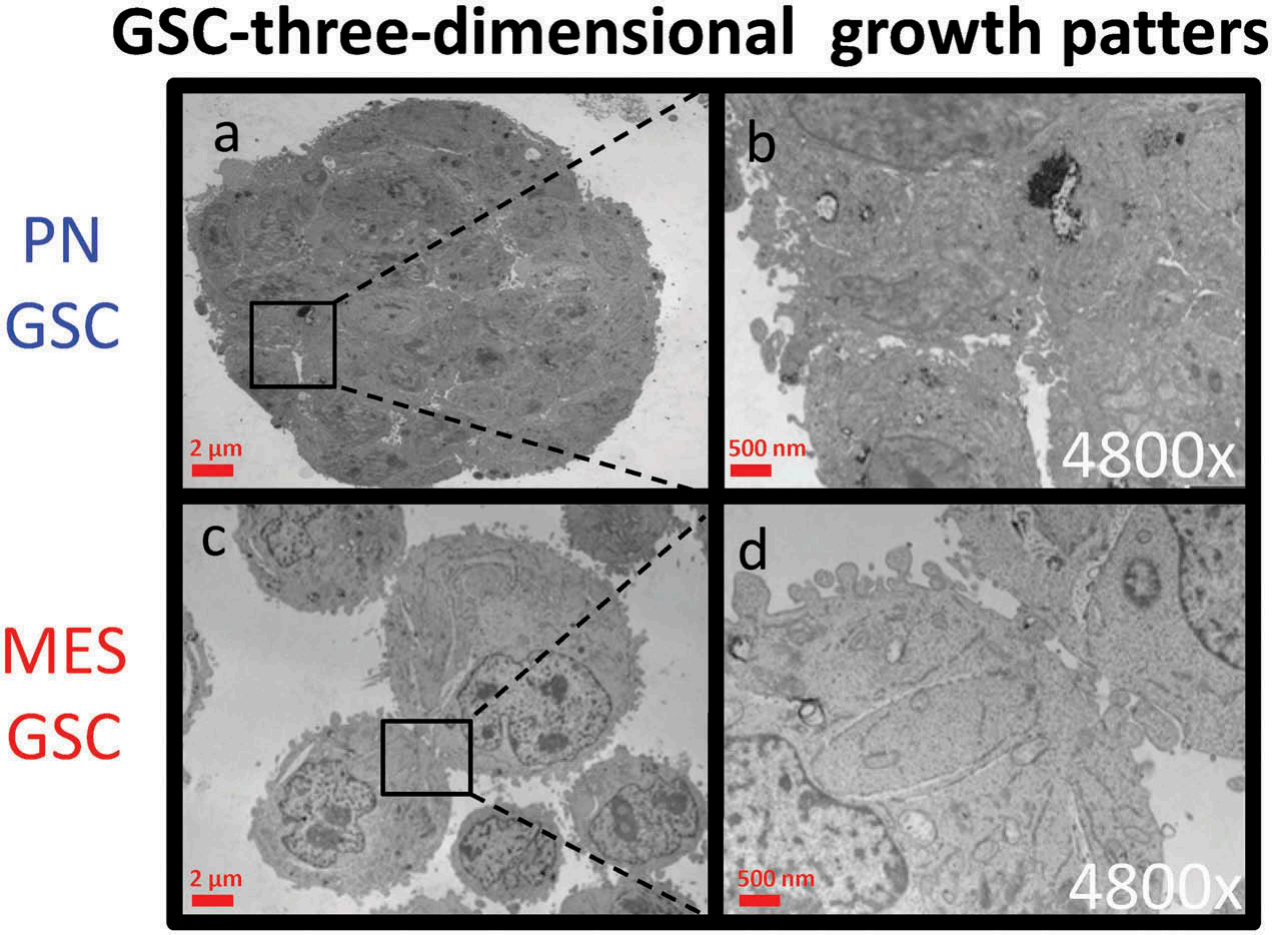
Transmission electron microscopy (TEM) of cultured GSC spheres. Morphological differences in three-dimensional growth pattern between PN (a-b) and MES GSC populations (c-d).

### The impact of induced GSC differentiation on the expression of vesiculation-related genes

**Figure 3.**
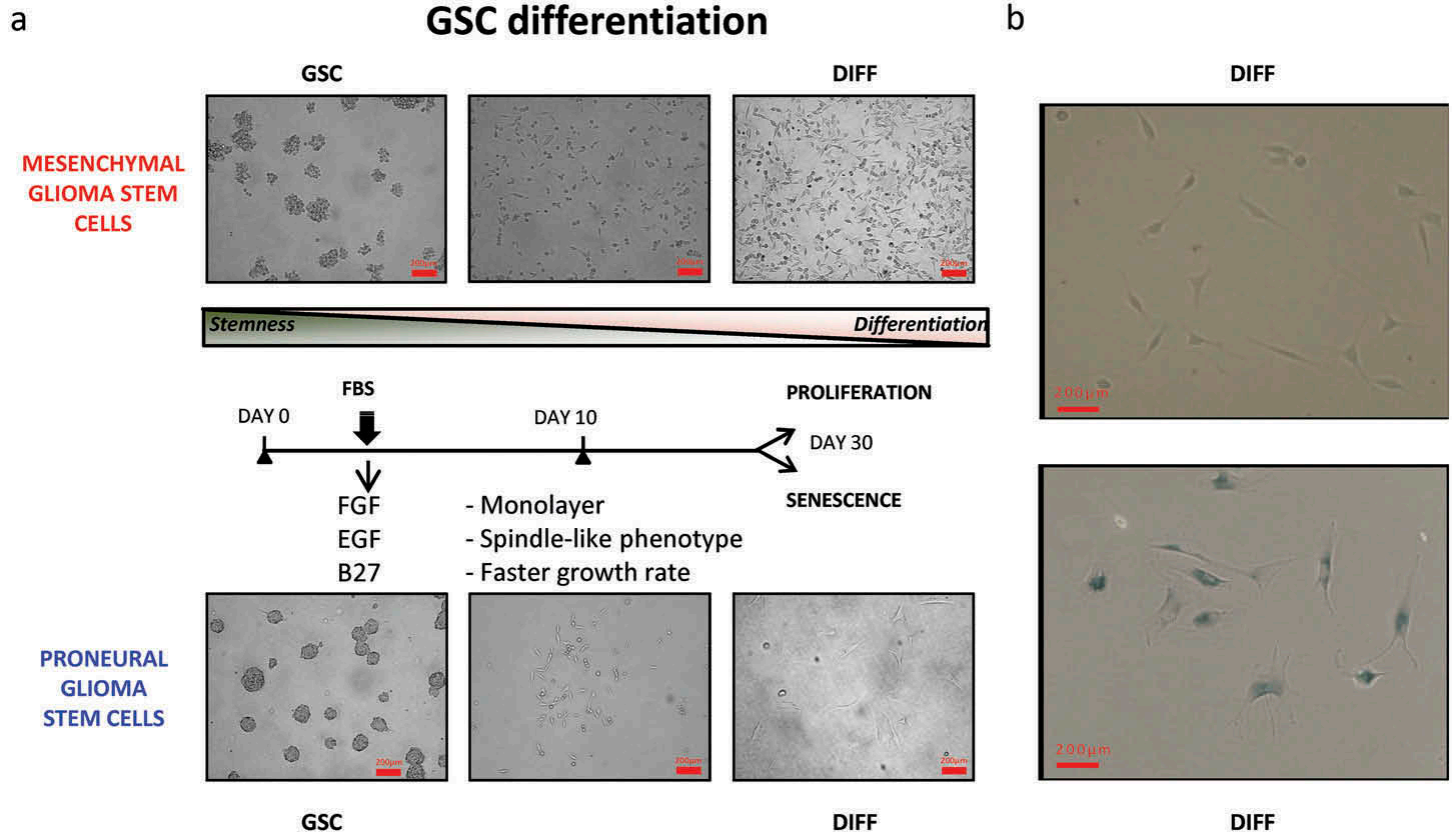
Cellular characteristics and phenotypic differentiation of proneural (PN) and mesenchymal (MES) GSCs in culture. Isolation and differentiation of proneural (PN) and mesenchymal (MES) glioma stem cells. (a) Schematic representation of GSCs differentiating into more committed cells in the presence of serum and after removal of stem cell growth factors (B27, FGF and EGF). After 30 days, PN differentiated cells (PN DIFF) enter senescence programme, while MES differentiated cells (MES DIFF) assume a more spindle-shaped phenotype, but continue proliferating. (b) Monitoring cell senescence by β-galactosidase assay (β-gal). While differentiated MES cells (top panel) remain β-gal-negative, their PN counterparts stain blue for β-gal (bottom panel), as described earlier [37].

Two defining features of GSCs consist of their ability to undergo self-renewal, and their capacity to populate the bulk of the tumour mass by GSC derived more differentiated cellular progeny [9]. Since little is known about the influence of these processes on the subtype-related intercellular communication mediated by EVs we set out to compare in this regard the EV profiles of GSCs representative of either PN or MES phenotype. For these experiments we chose cell lines GSC 157 and GSC 83, which exhibit PN and MES characteristics, respectively, and could be cultured under conditions known to favour either stemness or differentiation [9]. As previously characterized [16,37], the non-adherent PN GSCs formed tight multicellular spheres with extensive cell-cell contacts (Figure 2(a-b)), expressed high levels of the stem cell marker, prominin 1 (CD133), and almost no CD44 (Figure S3 A-B). They were also virtually negative for glial fibrillary acidic protein (GFAP), which is normally expressed by more mature astrocytes (Figure S3C-D). The sphere forming phenotypes were markedly different in the case of non-adherent MES GSCs, as indicated by both light and electron microscopy (Figures 3(a) and 2(c-d)). Thus, in keeping with earlier descriptions [16], MES GSCs formed grape-like clusters of loosely aggregated cells with the CD133^−^/CD44^+^/GFAP^low^ phenotype. In rare cases, clones of PN GSCs lost their characteristics over time, including tight intercellular connections. The related isogenic variants of these cells were established (as PMT1 cell line) in culture and these cells adopted some (but not all) features of the MES GSC phenotype (Figure S5A). These phenotypes and abilities to re-form neurospheres with distinct morphologies were also maintained when PN and MES GSCs were grown as adherent monolayers on laminin in the presence of the stem cell media [38].

**Figure 4.**
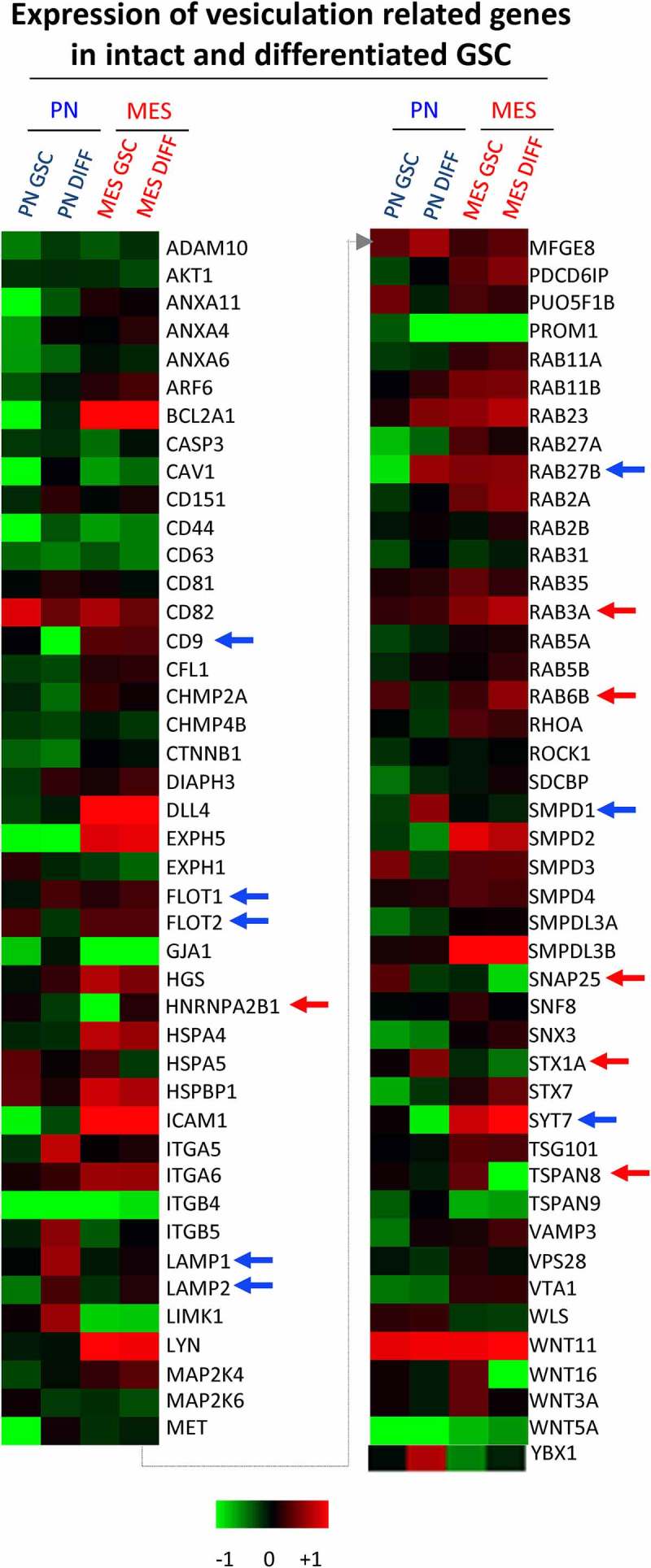
Quantitative PCR analysis of vesiculation-related genes reveals differences associated with GSC subtype and differentiation state. Customized RT2 targeted PCR arrays were used to evaluate the expression levels of vesiculation-related genes (vesiculome) in glioma stem cells (PN GSC and MES GSC) and their counterparts differentiated in the presence of serum (PN DIFF and MES DIFF). Upregulation and downregulation of notable genes involved in vesiculation were denoted by blue arrows for PN and red arrows for MES subtypes of GSC and DIFF cells.

The responses of both PN and MES GSCs to differentiation-inducing conditions were also distinct. Such a transition could be induced in GSC cultures by a well-characterized protocol involving the replacement of the serum-free, stem cell media containing recombinant EGF and FGF with media supplemented with high concentrations of serum (10%), no recombinant growth factors and under adherent growth conditions [9,39]. Indeed, both PN and MES GSCs when placed in such cultures for more than 10 days underwent remarkable phenotypic changes (Figure 3(a)) [40]. Initially, both PN and MES GSCs cells assumed spindle-like morphology, increased their growth rates and GFAP mRNA expression (Figure S3C-D). As differentiating cultures continued beyond 30 days, the fates of cells derived from either PN GSCs or MES GSCs (designated PN DIFF and MES DIFF, respectively) began to diverge dramatically. While PN DIFFs underwent terminal cessation of cell proliferation and stained positively for eukaryotic β-Galactosidase (B-Gal), a marker of cell senescence, MES DIFFs continued to proliferate as dense monolayers of spindle cells (Figure 3(b)). To explore the impact that this remarkable and subtype-related diversity may have on GSCs and their EV-mediated communication [16], we initially chose to assess the corresponding levels of the aforementioned set of 87 vesiculation-related transcripts (Figure 4). In this regard, notable changes were induced by differentiation in the case of PN GSCs including the upregulation of LAMP1, LAMP2, RAB27B, SMPD1 and FLOT1, while CD9, FLOT2 and SYT7 were downregulated (blue arrows). In MES GSCs, differentiation induced the upregulation of hnRNPA2B1, RAB3A and RAB6B, whereas STX1A, SNAP25 and TSPAN8 were downregulated in these cells (red arrows).

#### GSC subtype and differentiation influence the emission profiles of EVs

**Figure 5.**
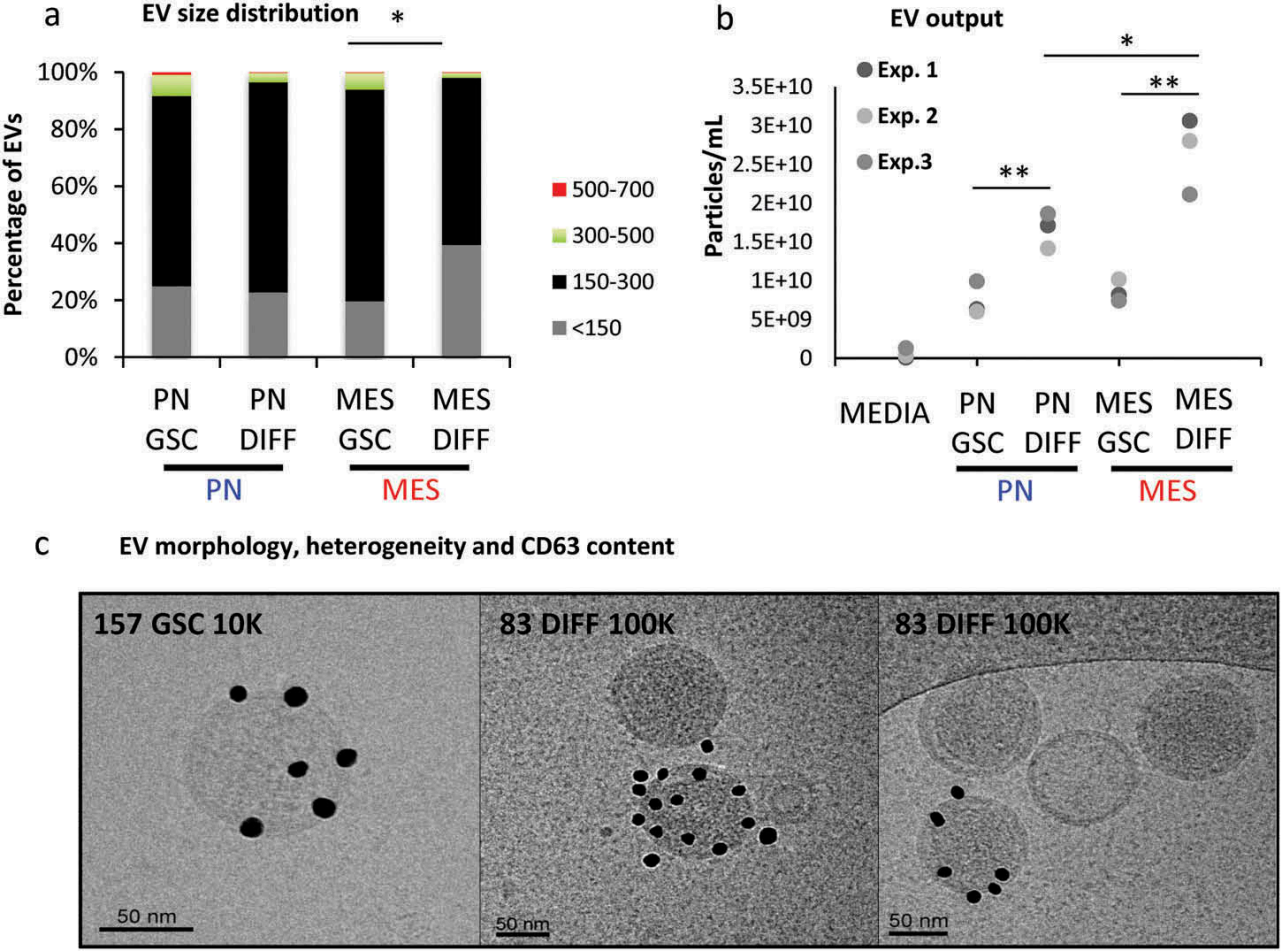
Characteristics of extracellular vesicles released by GSCs vary as a function of subtype and differentiation. (a) NTA analysis of EVs size distribution indicates that differentiated MES cells (MES DIFF) exhibit a significantly increased release of EVs smaller than 150 nm. (b) NTA profiles of the total particle numbers released by the GSC cell populations. Differentiation significantly increases the number of particles released by these glioma cells. (c) Cryo-TEM of EVs isolated from GSC and their differentiated counterparts illustrating the heterogeneity in EV size and CD63 immunogold labelling (dark particles). The images represent PN 10K pellet (left and middle panels) and MES DIFF 100K fraction (right panel), additional images are shown in Figure S3.

To assess more directly whether the aforementioned changes in gene expression are accompanied by the actual alteration in the cellular vesiculation profiles, EVs were purified from media conditioned for 3 days in the presence of PN GSCs, MES GSCs and their PN DIFF and MES DIFF counterparts. Following differential ultracentrifugation (DUC), as previously described [31], EV pellets were recovered after either 10,000 × g (10K) or 110,000 × g (100K) sedimentation, to separate ectosome/microvesicle-like and exosome-like EV fractions, respectively (Figure S6) [25]. Electron microscopy demonstrated the presence of EVs in the corresponding pellets with the expected [25] enrichment in small EVs, ranging from 100 to 300 nm diameter, in the 100k pellet (Figure 5(c), S4A-H and S7). The respective EV size distributions were also analysed using nanoparticle tracking (NTA) of unfractionated EV preparations and Cryo-TEM (Figure 5(a-c) and S7). While some larger EVs were, indeed, observed the majority of particles were found to be between 150 and 300 nm in diameter. Notably, a significant increase in the abundance of EVs under 150 nm in size was observed for cultured MES DIFF cells (approximately 40%; Figure 5(a)) and there was a significant increase of the total EV release following differentiation of both MES and PN cells (Figure 5(b)). Due to the fact that the 10K fraction appeared to represent different EV populations for PN and MES GSC cell lines and exhibited considerable intrinsic heterogeneity, we focused our subsequent analysis on the more uniform EV fraction sedimented under high centrifugation speed and reminiscent of exosomes (referred to as 100K; Figure S6).

**Figure 6.**
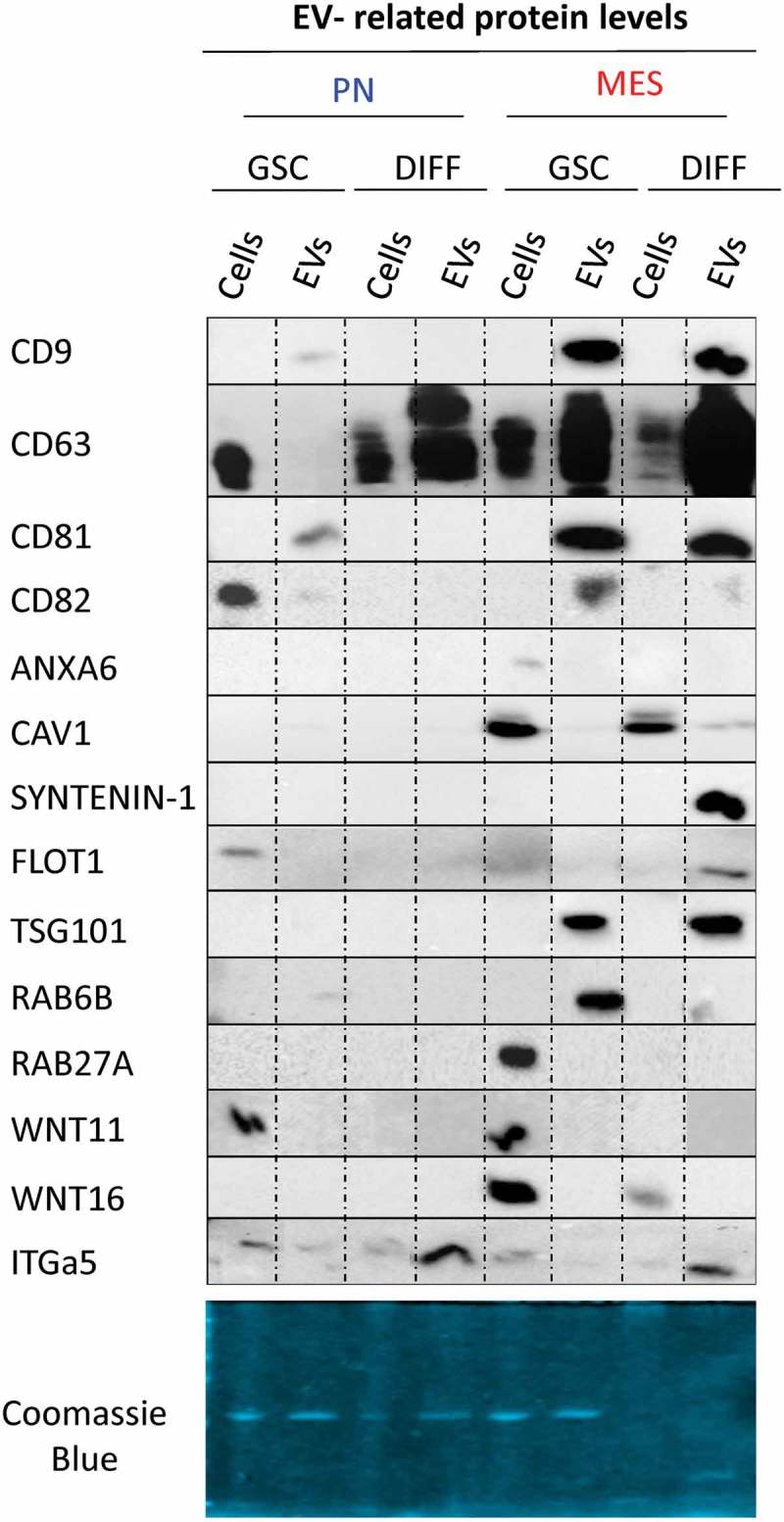
Distinct profiles of marker and cargo proteins reflect the impact of donor cell subtype and differentiation status on the biogenesis of extracellular vesicles. Immunoblotting for indicated proteins was performed using 2 μg of the protein preparation from 100K EV fraction isolated from conditioned media of the respective PN and MES GSCs and their corresponding differentiated progeny (DIFF). Of note are differences in expression of CD63, Syntenin-1, Flot1 and TSG101 (see text and supplementary figures).

Interestingly, GSC subtype and differentiation impacted both EV heterogeneity and molecular characteristics. Indeed, immunoblotting for a panel of EV markers and proteins related to vesiculome revealed stark differences between 100K EV fractions isolated from PN, or MES GSCs, and from their respective differentiated derivatives (PN DIFF and MES DIFF; Figure. 6, S5, S8 and S9). For example, the 100K fraction of small EVs released from PN GSCs exhibited only weak reactivity with antibodies against CD9, CD63 and CD82, with some signal for CD81. With exception of CD82 and CD63 these proteins were also undetectable in comparable amounts of the corresponding cell lysates. In contrast, 100K fraction of EVs released from MES GSCs was positive for CD9, CD63, CD81 and RAB6B, while weaker signal was recorded for CD82 and CAV1. These patterns changed markedly upon induction of cell differentiation with considerable upregulation of CD63 in the 100K fraction of PN DIFF-derived EVs and concomitant depletion of other markers. In the case of MES DIFF cells, we observed strong expression of CD9, CD63, CD81, Syntenin-1, Flotillin-1 and TSG101, while RAB6B and CD82 were not readily detectable (Figure 6).

This global quantitative marker distribution does not preclude the existence of distinct EV subsets with alternative characteristics within the GSC secretomes. For example, while the total levels of CD63 in the EV fraction of PN GSCs were exceedingly low, as measured by Western blotting (Figure 6), subsets of CD63-positive EVs were readily detectable in this material using immuno-Cryo-TEM (Figure 5(c)) or nano-flow cytometry (data not shown). In this case, the Western blotting results were confirmed with several alternative gel loading normalization schemes according to either Coomasie blue stain (Figure 6), protein quantification or NTA counts (Figure S9A, top and bottom panels, respectively), and through the use of two different validated monoclonal anti-CD63 antibodies. This included H5C6 clone (Figure S9B) and EPR5702 clone, the former revealing a faint CD63 band in PN GSC EVs (Figure 6 and S9B). Nonetheless, our results suggest that the subtype and differentiation states of GSCs are associated with remarkable rearrangements in molecular features of EVs released by these cells into their milieu.

#### Evolution of proneural GSCs in culture results in changes of their EV profile

Due to the evolving nature of GBM [7], MES phenotype may often emerge at relapse from an earlier PN-type lesion [8]. To examine whether the occurrence of such a change in isogenic settings may impact vesiculation of cultured GSCs, we employed the PMT1 cell line (described earlier), which spontaneously originated from PN GSCs, but lost some of the features associated with the PN phenotype and adopted more MES-like characteristics (Figure S5A-B). Indeed, EVs isolated from these cells acquired higher CD9 and RAB27A protein expression relative to their PN parental cells. Also, the comparison of vesiculation-related transcripts expressed by PMT1 and PN GSC lines revealed a significant upregulation of CD44, BCL2A, MAP2K6, LIMK1 and several other genes, while mRNA for POU5F1B, LAMP2, GJA1 and VAMP3 were downregulated (Figure S5C). Collectively, these observations suggest that pathways defining molecular GSC subtypes and differentiation modulate the quantitative, qualitative and molecular aspects of the EV emission profile [39] in glioblastoma initiating cells.

#### GSC subtype and differentiation regulate the EV proteome

**Figure 7.**
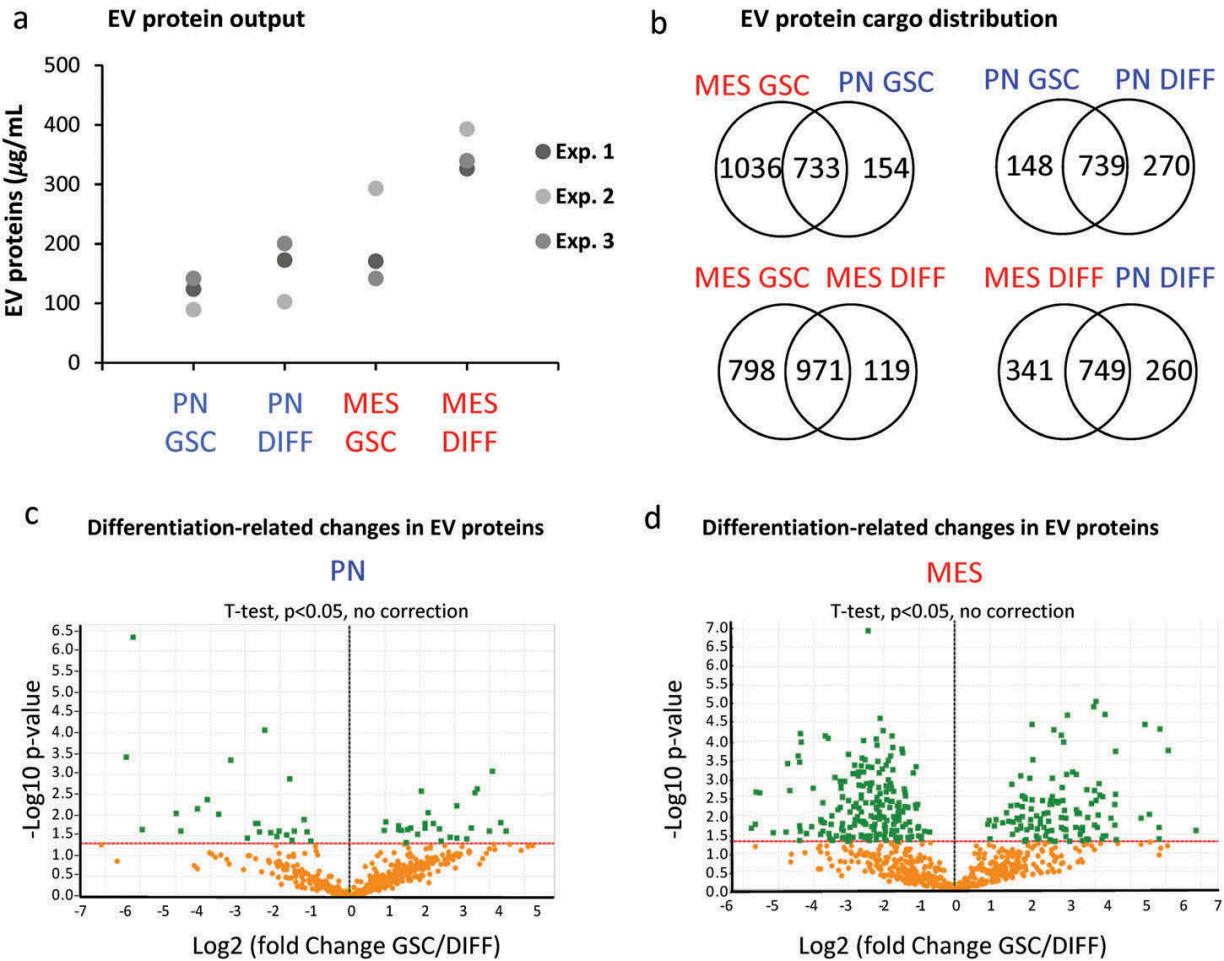
The impact of GSC subtype and differentiation status on the EV proteome. (a) Protein quantification in EV isolates from comparable GSC and DIFF cultures of PN and MES glioma cell lines. MES derived EV preparations contain higher total protein content than that of PN EVs. (b) Venn diagram of common and unique EV proteins identified by mass spectrometry in preparations of conditioned media from the indicated cell lines. (c-d) Differential expression of proteins expressed in GSC versus DIFF cells within PN and MES cell subtypes, respectively. MES glioma cells contain a greater number of proteins relative to PN cells. Coordinates: x axis = log2(fold-change) (GSC/DIFF), y axis = −log10(P value). The horizontal line indicates P value = 0.05. Data represent results of three independent experiments pooled together.

**Figure 8.**
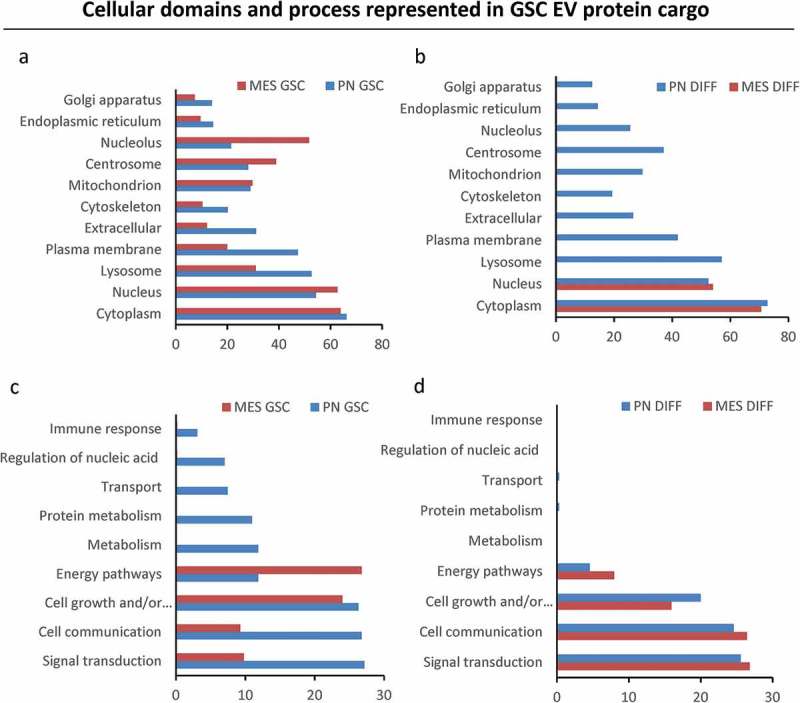
Gene ontology analysis of extracellular vesicle proteomes across subtypes and differentiation states of donor GSCs. GO analysis was performed using FUNRICH database. EV cargos of the indicated GSC populations differ with regards to their cellular compartment (a) and molecular functions (b).

We reasoned that numerical and molecular shifts in the characteristics of GSC-derived EVs under different growth conditions likely reflect a more fundamental change in the vesiculation process. To assess the nature and plausible consequences of this regulation, we developed proteomic profiles (LC-MS/MS) of the 100K EV fraction purified from conditioned media of PN GSC and MES GSC, as well as PN DIFF and MES DIFF cells. At least four different peptides were used for representation and quantification of individual proteins. Interestingly, while the total amount of proteins present in these preparations was comparable (Figure 7(a)), albeit higher in MES cells, the protein compositions were markedly different between EV pools released from different donor cells (Figure 7(b-d)). For example, 733 proteins were common for EVs from MES and PN GSCs, but 1036 and 154 were unique to these respective donors. Similar comparisons depicted as Venn diagrams indicate the existence of proteins uniquely associated with reprogramming of the EV cargo by GSC subtype, differentiation and both (Figure 7(b)). We have also analysed top 50 proteins enriched in each cell-specific EV fraction. These comparisons indicate a degree of similarity (but also differences) between 100K EV fractions from each cellular source, but a clear separation between GSC and DIFF growth conditions (Tables S2). We also quantified the enrichment of EV proteins during GSC to DIFF transition for both PN and MES lines as depicted in the volcano plot (Figure 7(c-d)). The levels of several proteins were found to change by a factor of > 2-fold and were both significant (P < 0.05) and subtype specific. For example, for PN GSC line the differentiation process induced an increase in EV-associated ALDR, Galectin-1, Sorcin, TSP1, HSPB1, while similar treatment of MES cells lead to enrichment of IQGAP1, PA2G4, PELP1, HNRNP family, NPM1, EIF3. We then explored the functional aspects of this enrichment using Gene Ontology (GO) tools for term enrichment analysis focusing on subcellular localization (Figure 8(a-b)) and biological process (Figure 8(c-d)). This audit yielded further support of EV subtype specificity in that PN GSCs emitted EVs containing proteins strongly associated with plasma membrane and extracellular domains, while EV proteins derived from MES GSCs were largely assigned to nuclear compartments (Figure 8(a)). Differentiated GSCs emitted EVs derived mainly from the cytoplasm but there were also differences between PN and MES subtypes (Figure 8(b)). Similarly, auditing EV proteome for molecular function suggested a link to signal transduction and cell communication in the case of PN GSC, PN DIFF and MES DIFF cells, while EVs from MES GSCs were enriched for proteins linked to nucleic acids and proteins metabolism (Figure 8(c-d)).

**Figure 9.**
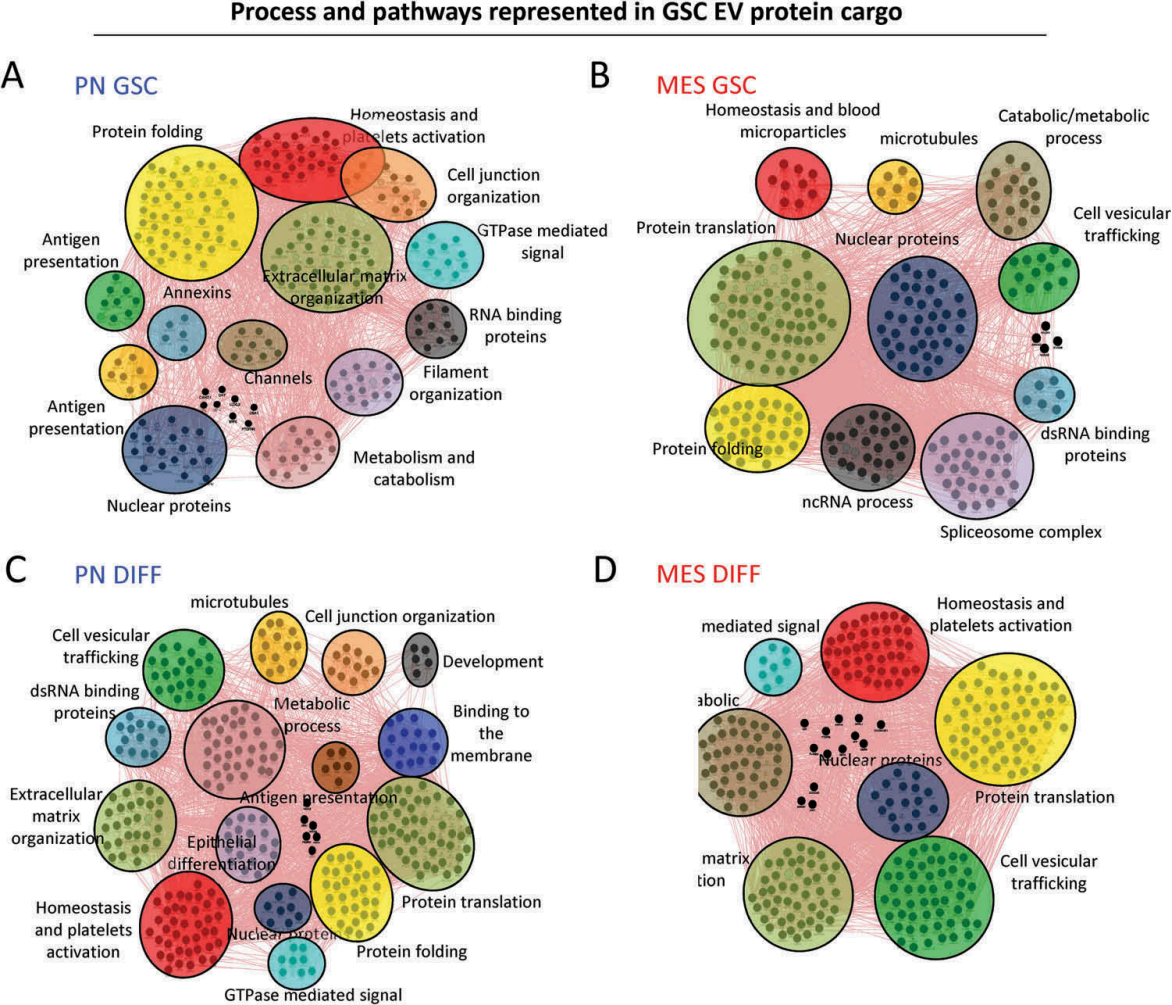
Cytoscape representation of changes in functional clusters of extracellular vesicle-associated proteins released from mesenchymal and proneural GSCs in their undifferentiated and differentiated states. GO analysis was performed using geneMANIA software. Network of proteins in PN (a) and MES (b) GSC-derived EVs and those detected in PN (c) and MES (d) DIFF-derived EVs. The clusters and their constituent proteins dramatically changed between subtypes and differentiation states of glioma cell subsets.

Using the GeneMANIA application of Cytoscape software [42], we were also able to build interactive networks of proteins pathways for GSC (Figure 9(a-b)) or DIFF cells (Figure 9(c-d)), both PN and MES subtypes. The results show that PN GSC-derived EV proteins mainly cluster around networks involved in proteins folding and in cellular/extracellular matrix organization (Figure 9(a)), while MES GSC-derived EVs are enriched for proteins that play a role in protein translation and nucleic acids processing with an increase of proteins derived from the nucleus (splicesome complex, nuclear proteins) (Figure 9(b)). Protein networks were also affected by the differentiation status of glioma cells. Thus, PN DIFF-derived EVs exhibited increased representation of proteins involved in metabolic process, protein translation, hemostasis and platelet activation (Figure 9(c)). Similarly, MES DIFF-derived EVs carried proteins representative of metabolic process, hemostasis and platelet activation (Figure 9(d)). Notably, cargo of MES DIFF EVs contained markedly increased content of proteins involved in extracellular matrix organization and vesicular trafficking compared to their GSC counterparts. Together the results suggested a potential for different effect of EVs in recipient cells when their cargo is transferred.

#### GSC subtype and differentiation programmes influence the biological activity of glioma-derived EVs

**Figure 10.**
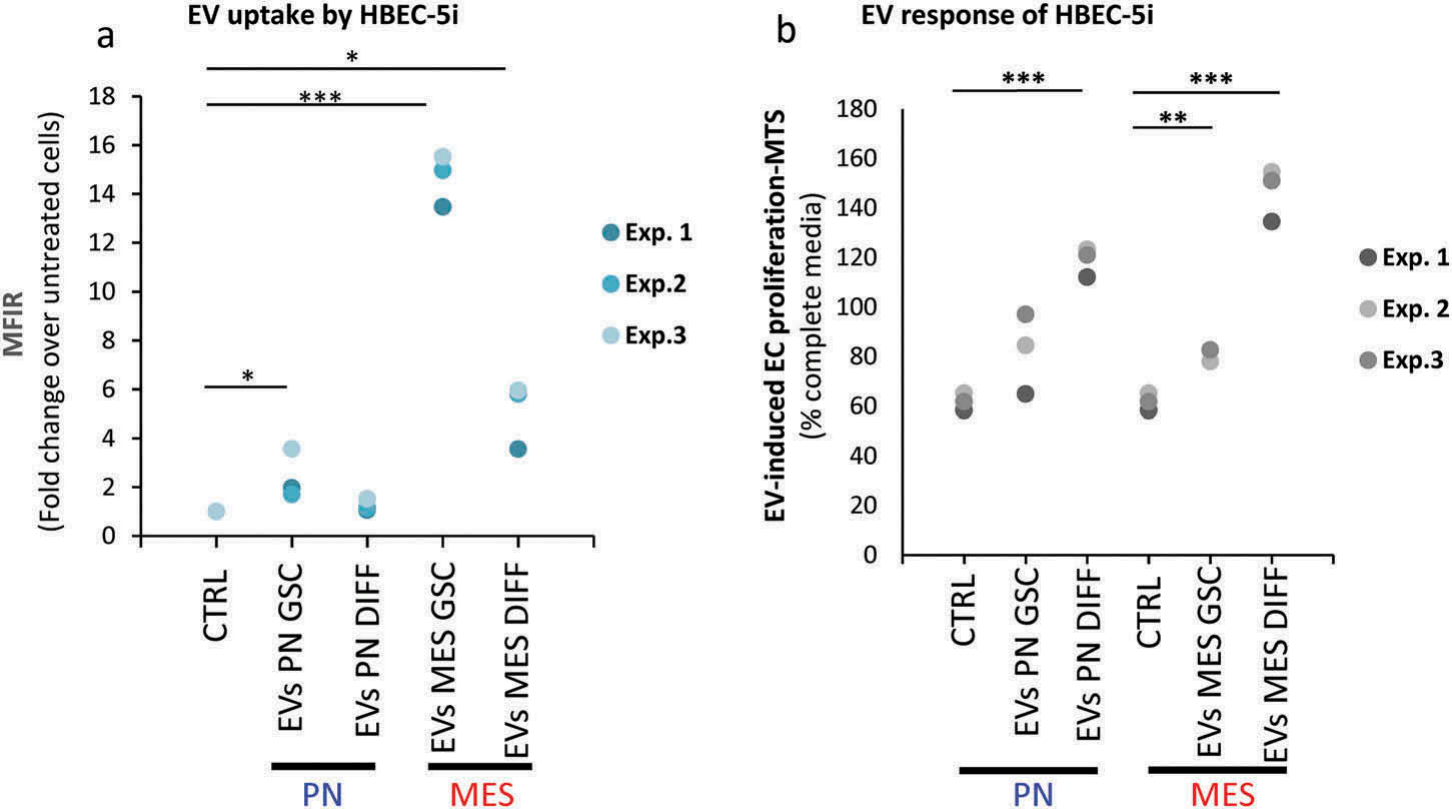
Differential uptake and endothelial stimulating activity of EVs as a function of donor GSCs subtype and differentiation status. Endothelial cell (HBEC-5i) uptake of, and proliferative response to EVs, as measured by FACS and MTS, respectively. (a) PKH26-labelled EVs were incubated with HBEC-5i recipients and the fluorescence transfer was quantified by flow cytometry. HBEC-5i preferentially uptake EVs from MES GSC. (b) The 100K fractions of indicated EV isolates were used to stimulate HBEC-5i cell proliferation (MTS). The cellular responses are particularly notable in the case of EVs from DIFF glioma cells, especially of the MES subtype.

EV emission not only reflects the state of cancer cells, but also connects them with other cellular populations, of which vascular endothelium plays a unique role in GBM [13,18,22,41]. Since one aspect of this interaction may involve EV internalization by recipient endothelial cells [34,43,44] we tested whether such uptake for vesicles released from different donor cells (PN GSCs, MES GSCs, PN DIFF, MES DIFF) is equivalent or different. To accomplish this, 30 μg/mL of glioma EVs were labelled with fluorescent dye (PKH26) and incubated with human brain endothelial cells (HBEC-5i) for 16 h, followed by testing for cell-associated fluorescence using FACS (Figure 10(a) and S10). While EVs from all GSCs donors were taken up by endothelial cells, this process was remarkably more efficient in the case of EVs produced by MES GSC and MES DIFF cells, which generated the uptake signal that was, respectively, 7- and 2-fold higher than that evoked by similar numbers of EVs released from PN GSCs or PN DIFF donors, which are poorly internalized by endothelial cells.

Although this observation may suggest different biological (vascular) activities of EVs derived from either PN or MES GSCs, this aspect is ultimately influenced by the molecular cargo where we previously noted significant differences. Given the fact that endothelial cell proliferation is both an essential component of angiogenesis and a hallmark of GBM vasculature [20], we asked whether GSC EV transfer plays a role in proliferative responses of HBEC-5i cells in culture. HBEC-5i cells were incubated for 6 days with either growth media containing 10% FBS, starvation media (1% FBS), or with starvation media supplemented with EVs from various donor cultures at the optimized concentration of 30 μg/mL. As revealed by MTS assays (Figure 10(b)), EVs were able to significantly increase endothelial cell proliferation/survival, as compared to vehicle control (PBS), often above the level observed in optimized growth media. Somewhat surprisingly, this effect was most pronounced for EVs derived from MES DIFF cells, followed by PN DIFF donors. These observations suggest that while the ability of glioma EVs to interact with endothelial cells is dependent on GSC subtype and differentiation, the EV uptake does not predict the related mitogenic responses. Overall our study highlights the role of heterogeneous GCS subtypes and states in the regulation of the EV-mediated intercellular communication processes.

## Discussion

Our study brings to the fore several novel observations. First, we provide evidence that molecular subtypes of GBM impinge upon genes regulating the biogenesis and cargo of different EV populations. In this sense, genetic driver mutations that define GBM subtypes could be seen as regulators of not only cellular phenotype but also EV mediated intercellular communication [17]. This is important as molecular profiling has revolutionized the outlook at GBM, effectively dividing it into several distinct disease states in which the networks of cellular connectivity are likely to be markedly different [19]. Second, this molecular diversity is reflected (but not copied) in the case of GSCs [16], the vesiculation profiles of which are also influenced by molecular subtype, including emission rates, phenotypes, EV proteome, uptake and biological activity. Third, we demonstrate that, in addition to hardwired oncogenic and cellular determinants of the GSC subtype, also epigenetic ability of these cells to differentiate impacts molecular and functional EV characteristics. Fourth, properties of donor cells define the ability of EVs to interact with endothelial cells and influence their characteristics relevant to angiogenesis.

Angiogenic effects of EVs are of interest as vascular abnormalities are both common and pathognomonic in GBM [15,20]. Forces that influence the vascular compartment in GBM include microenvironmental queues, such as hypoxia, but are also strongly dependent on direct effects that oncogenic pathways exert upon the expression of angiogenic mediators such as vascular endothelial growth factor (VEGF), coagulation factors and cellular vesiculation [18]. This may separate the nature of vascular events in GBM from physiological angiogenesis and explain the surprisingly unsatisfying performance of potent VEGF inhibitors in this disease [45]. In this light, a better understanding of EV roles in GBM angiogenesis is especially pressing, as EVs carry a complex repertoire of signalling cues, including bioactive lipids, proteins, and nucleic acids, all of potential relevance to blood vessel regulation [23,46]. Cancer-related roles of EVs may include transfer of bioactive oncogenes to endothelial cells [44,47], microRNA-dependent changes in vascular growth patterns [48], regulation of vascular permeability [49,50] and other effects [22,41,51]. GSC are uniquely positioned as contributors to tumour-vascular interactions in GBM due to their physical proximity and dependence on the capillaries [13] as well as heightened production of VEGF [14], VEGF-containing EVs [29] and other vascular activities [52].

While the related literature focuses on the angiogenic commonalities of GSCs, our study highlights their heterogeneity. We document stark differences in EV biogenetic programmes across large number of GSC lines examined for their vesiculome, and a degree of convergence of these patterns on PN and MES transcriptional subtypes. We investigated the consequences of this diversity in a greater depth using representative PN and MES cell lines, as well as an isogenic GSC variant (PMT1 cells) that originated from PN cells, but spontaneously acquired some of the properties associated with the MES phenotype. Using these model cell lines, we uncovered remarkable differentials in the type, proteome and biological activity of the related EVs and the impact of cell differentiation on these properties. While proteomes of GSC populations and their EVs have been previously studied [28] and their markers analysed [53], out work points to epigenetic plasticity of these properties across cellular differentiation programmes, different for PN and MES GSC subtypes.

Notably, earlier reports highlighted largely the prominent contribution of CD133^+^ GSCs to the release of soluble angiogenic factors such as VEGF relative to more committed, CD133^−^ GBM bulk cells [14]. In contrast, our studies reveal a robust EV-dependent endothelial cell stimulating activity of differentiated gliomas cells, including CD133^low^/GFAP^high^ PN DIFF and especially the 100K (exosome-like) fraction of EVs from CD133^−^/GFAP^high^ MES DIFF cell population. This may suggest that angiogenic circuitry in GBM may include different complementary inputs from GSCs and their differentiated progeny, each contributing different amounts of either VEGF (growth factor)-dependent or EV-dependent activities, the latter of which were never therapeutically addressed [44]. The relation of these events to molecular subtypes of GSC (and GBM) is illustrated by a striking preference with which the same endothelial cells uptake EVs emanating from MES but not PN cells. It remains unclear what molecular components of EVs define their uptake or endothelial stimulating activity, but we did not observe any discernable contribution of VEGF or VEGF signalling pathway to these responses (data not shown).

Although our experiments demonstrate, in principle, that the heterogeneity of GSC subtypes compounded by their differentiation states may shape the molecular repertoire and biological activity of EV populations, much remains to be studied. For example, the molecular mechanisms involved at different steps of EV regulation in GBM cells (EV biogenesis, cargo assembly, uptake and angiogenic activity) are of great interest and are presently unknown. Whether and how different pathways of GSC differentiation have an impact on EV profiles of the resulting divergent cell types e.g. GSC-derived astrocytic, endothelial-like [54] or pericyte-like GBM cell populations [14] is also open to question. The nature and context of EV interaction with angiogenic or quiescent endothelial cells is poorly understood outside of surrogate growth or migration assays. Likewise, the generality of EV properties across larger panels of GBM and GSC isolates or subtypes, and their representation in intact tissues are still to be addressed more fully, and are currently under study. Moreover, therapy-induced evolution of GSCs [55] is likely to impact EV profiles, as we recently documented [56], while intra-tumoural interactions of different GSC population may also involve EV-dependent signals [28].

Our study adds a new dimension to these multifaceted efforts by exploiting the process of cellular vesiculation as a regulatory target of not only oncogenic pathways [18,34], but also cellular stemness, molecular subtype and epigenetic modulation [47,57]. The intricacy of this emerging EV landscape could help refine the approaches to develop EV biomarkers in GBM and may reveal new therapeutic attack points in this incurable disease.

## Geolocation

McGill University, RIMUHC Montreal, QC, Canada.
