# Supplementary figures and images for: Molecular subtypes and differentiation programmes of glioma stem cells as determinants of extracellular vesicle profiles and endothelial cell-stimulating activities
Source: J Extracell Vesicles. 2018 Jul 17;7(1):1490144. doi: 10.1080/20013078.2018.1490144 (PMC6052423; doi:10.1080/20013078.2018.1490144)

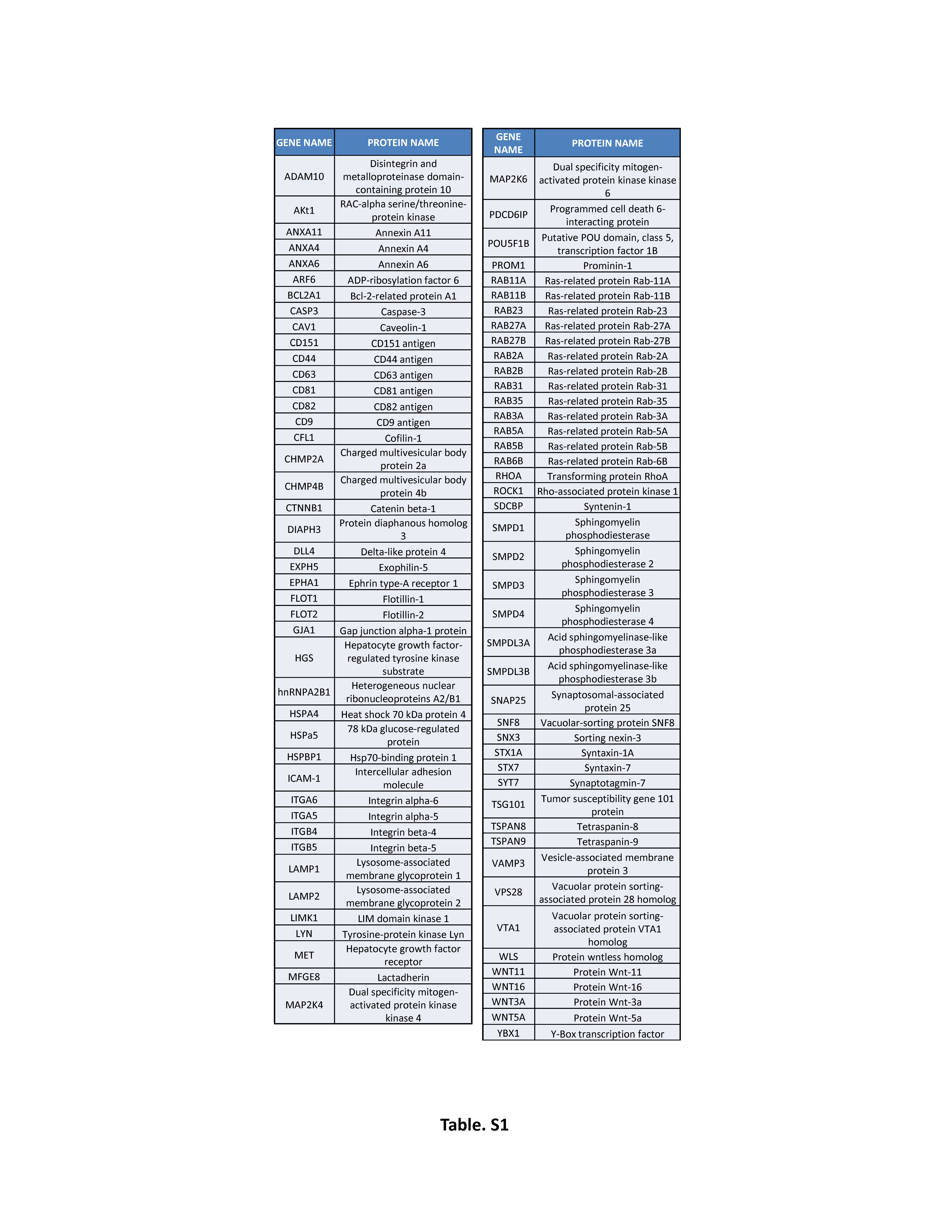

Supplement: Supplemental Material [file ZJEV_A_1490144_SM0503.zip › 0Spinelli_JEV_FINAL_new_graphs_jr_Page_12.tiff]

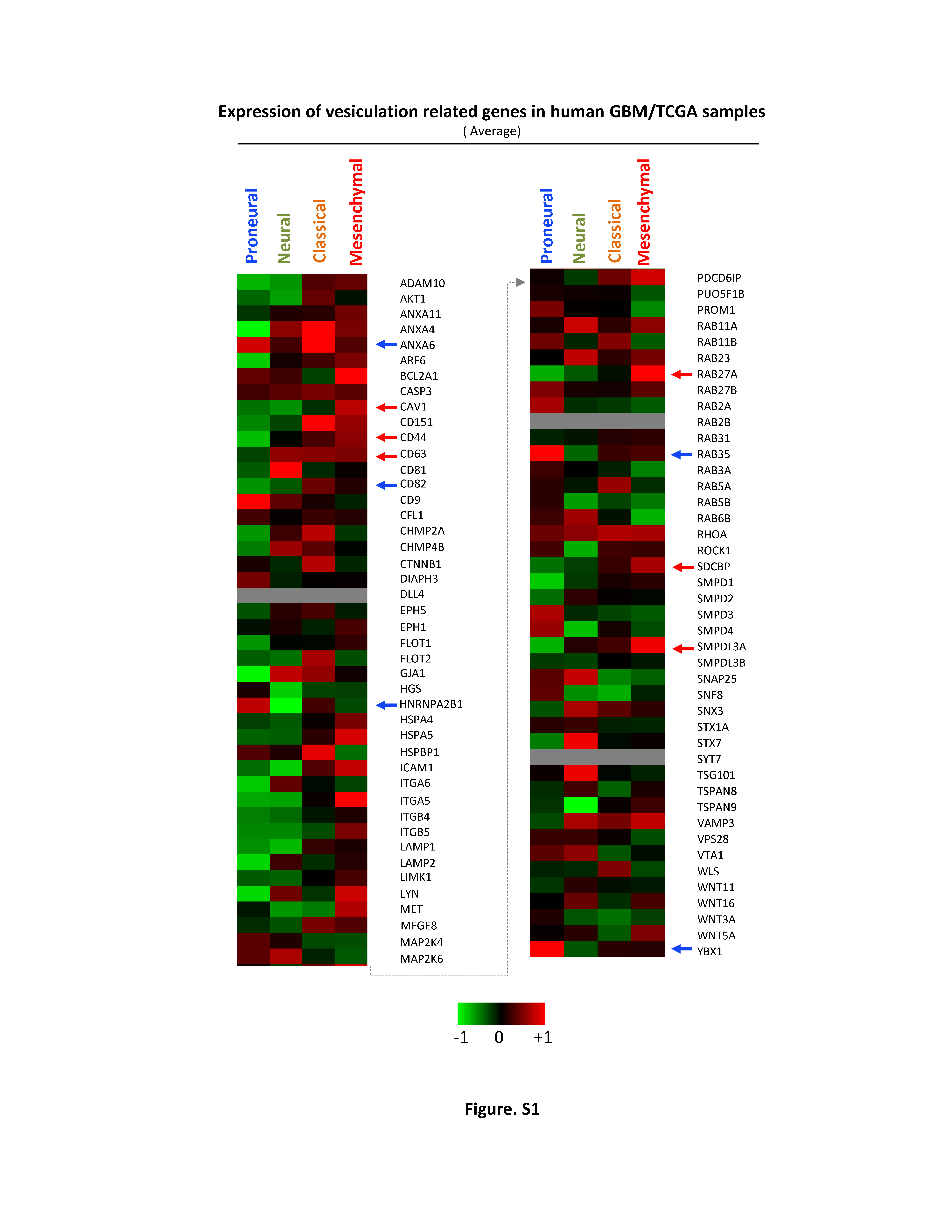

Supplement: Supplemental Material [file ZJEV_A_1490144_SM0503.zip › 0Spinelli_JEV_FINAL_new_graphs_jr_Page_13.tiff]

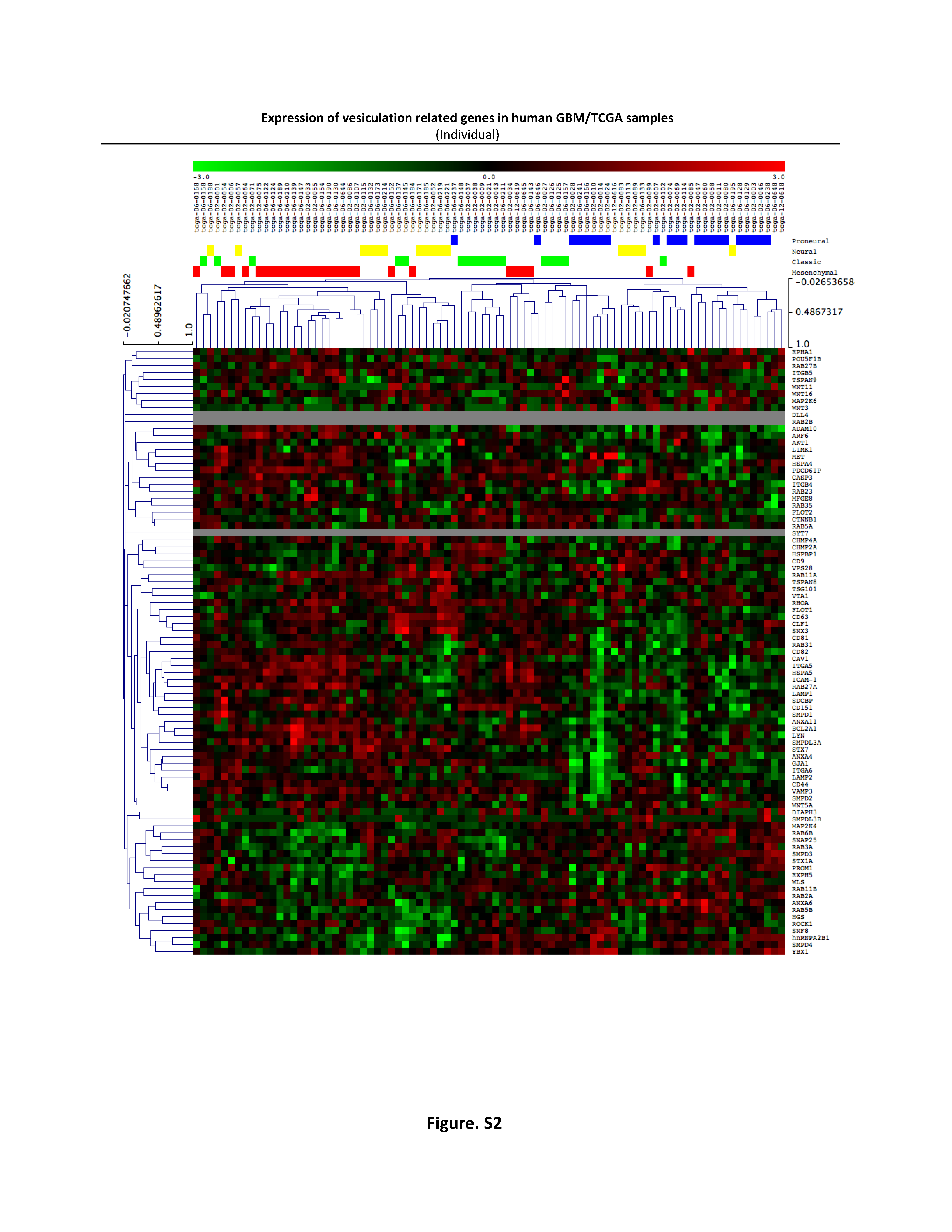

Supplement: Supplemental Material [file ZJEV_A_1490144_SM0503.zip › 0Spinelli_JEV_FINAL_new_graphs_jr_Page_14.tiff]

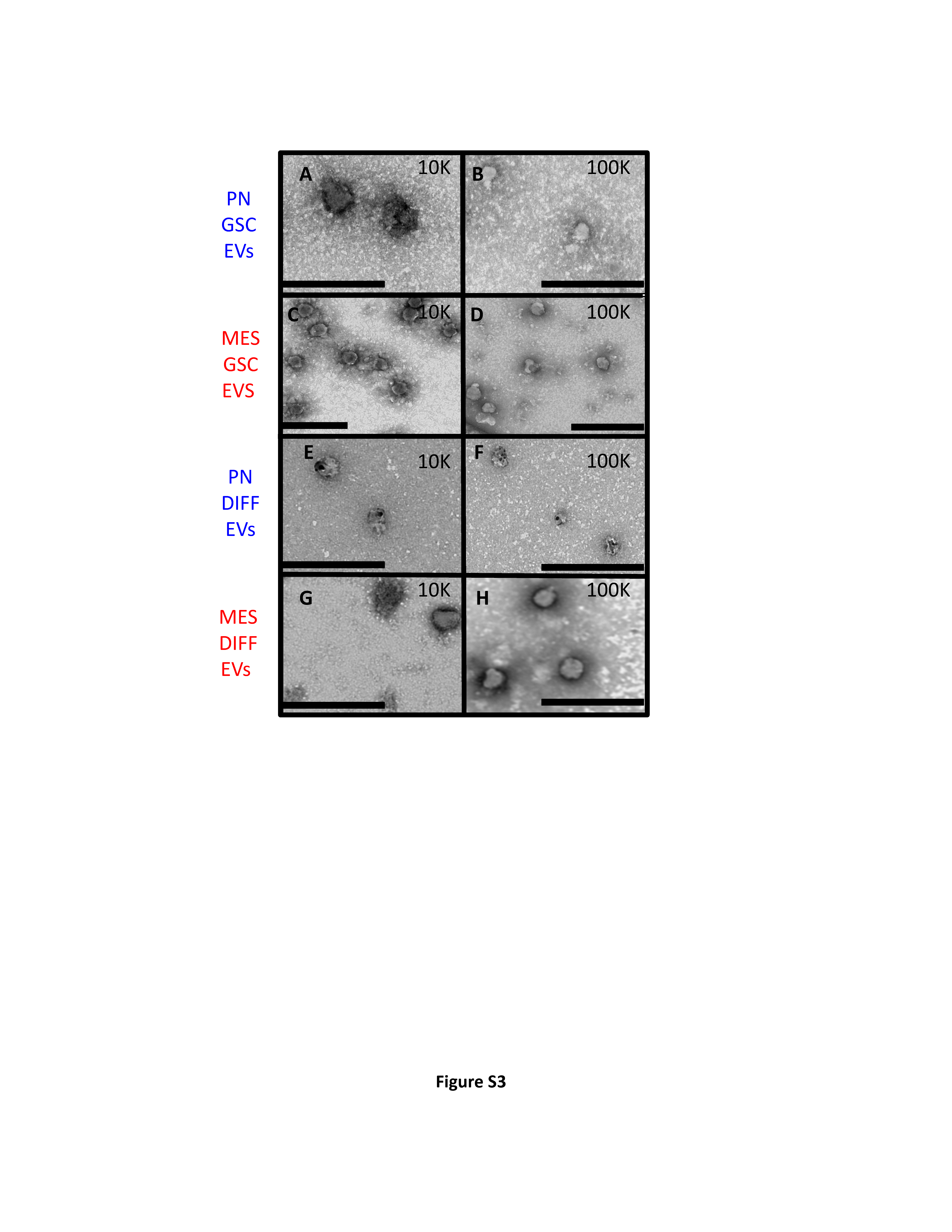

Supplement: Supplemental Material [file ZJEV_A_1490144_SM0503.zip › 0Spinelli_JEV_FINAL_new_graphs_jr_Page_15.tiff]

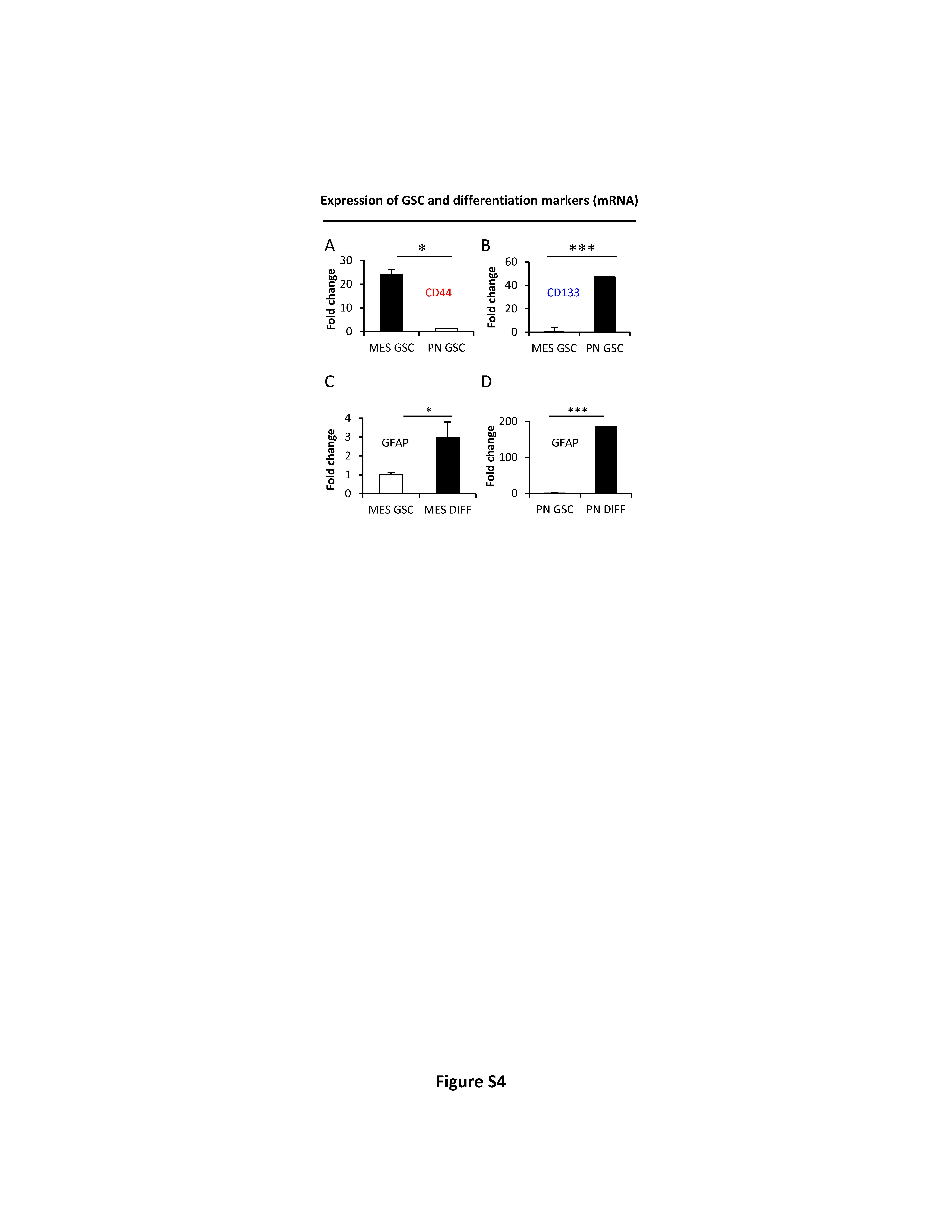

Supplement: Supplemental Material [file ZJEV_A_1490144_SM0503.zip › 0Spinelli_JEV_FINAL_new_graphs_jr_Page_16.tiff]

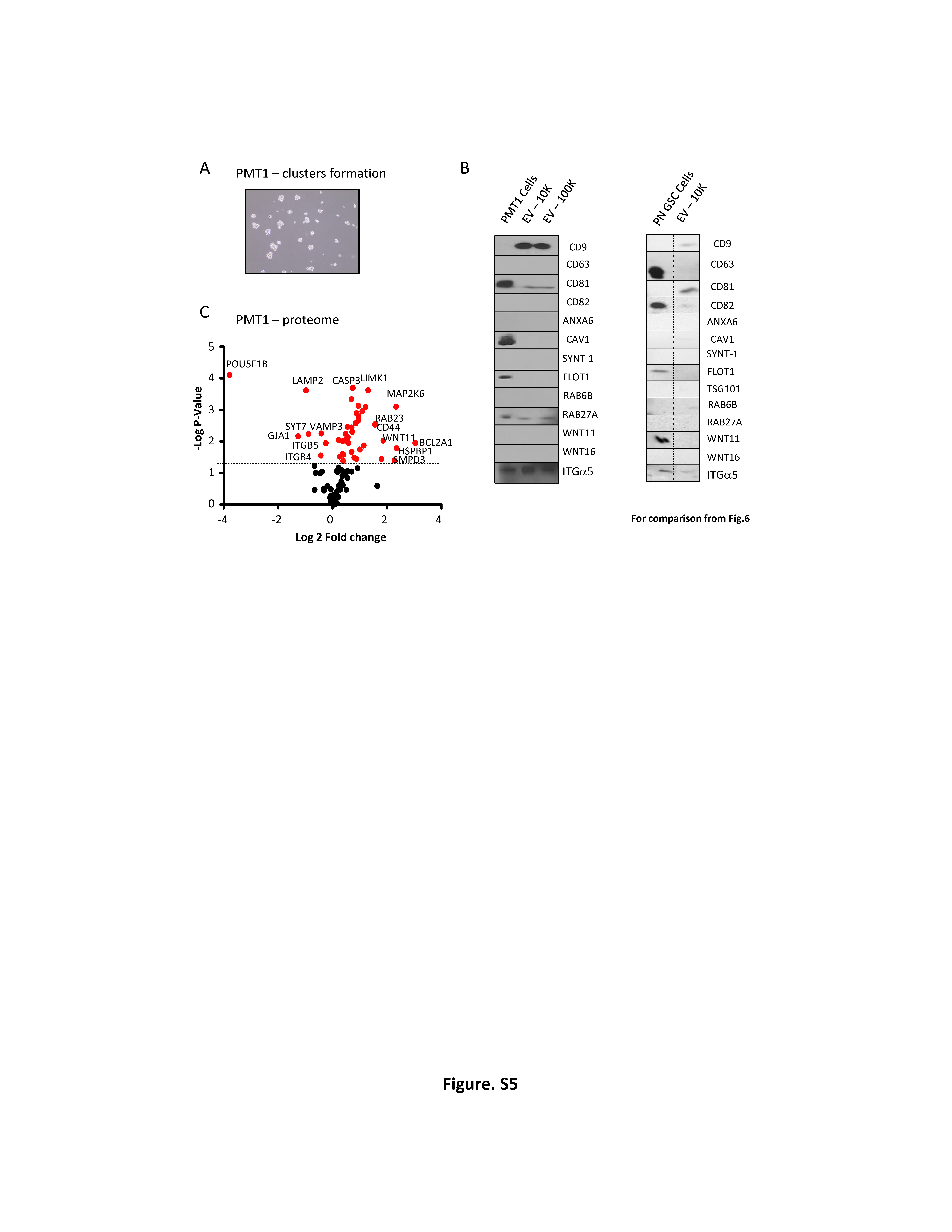

Supplement: Supplemental Material [file ZJEV_A_1490144_SM0503.zip › 0Spinelli_JEV_FINAL_new_graphs_jr_Page_17.tiff]

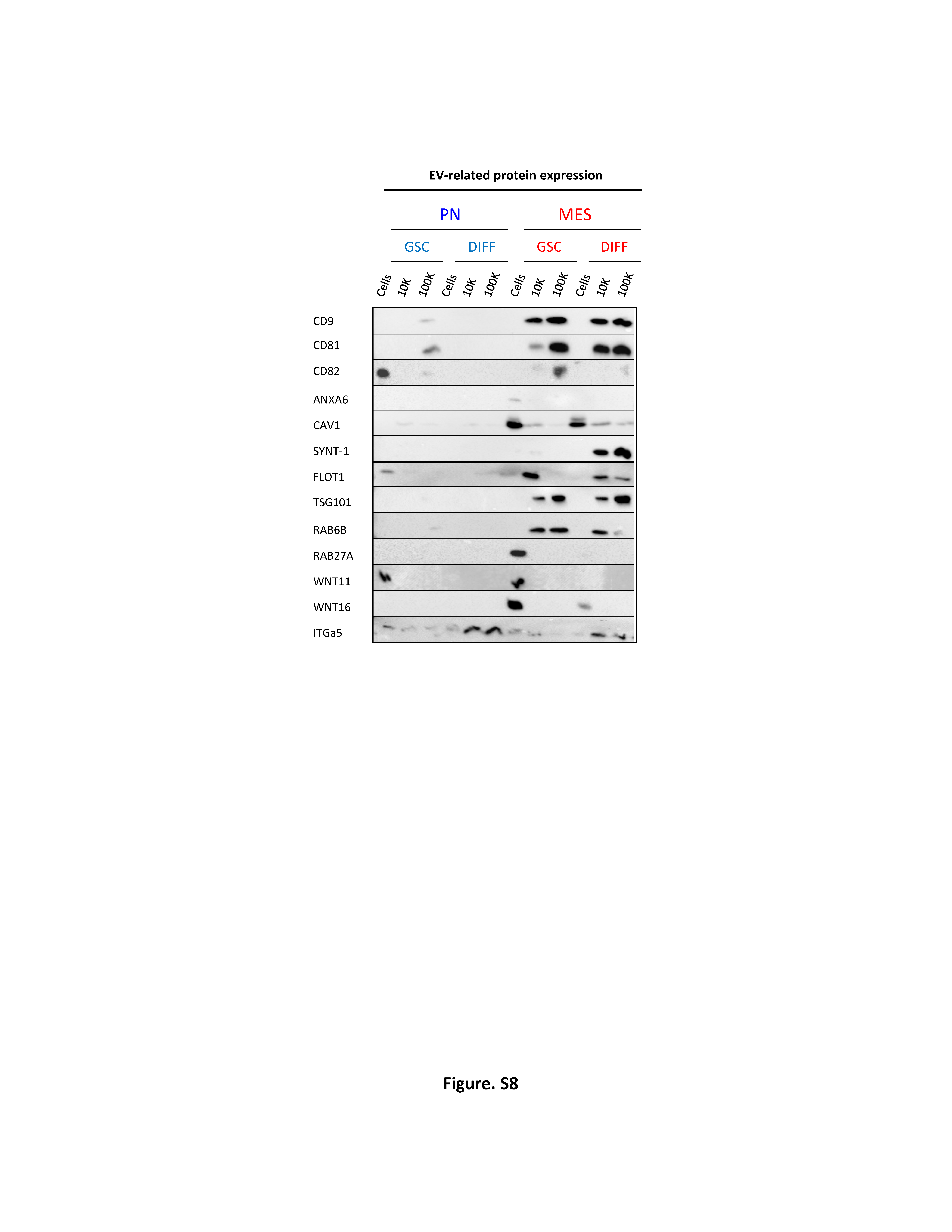

Supplement: Supplemental Material [file ZJEV_A_1490144_SM0503.zip › 0Spinelli_JEV_FINAL_new_graphs_jr_Page_20.tiff]

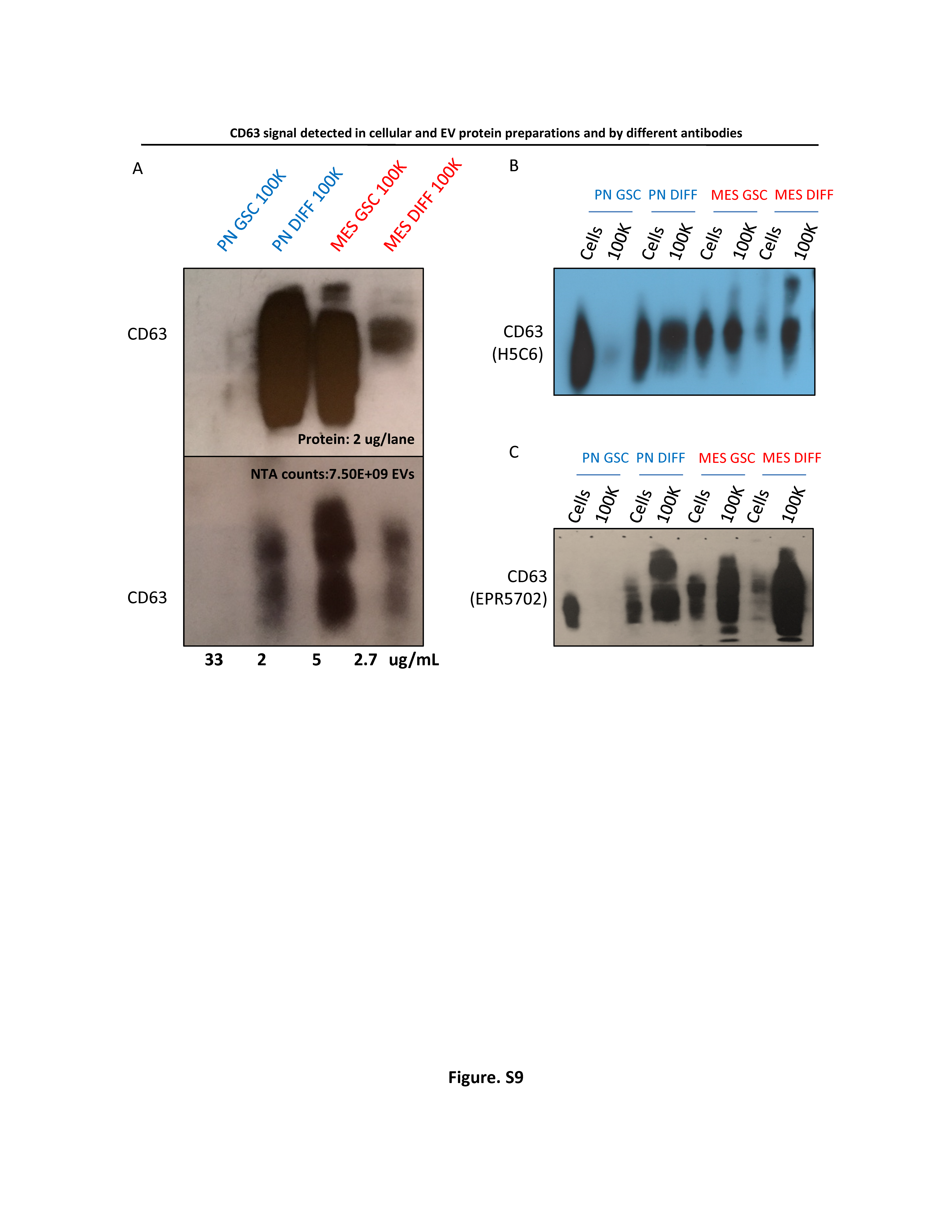

Supplement: Supplemental Material [file ZJEV_A_1490144_SM0503.zip › 0Spinelli_JEV_FINAL_new_graphs_jr_Page_21.tiff]

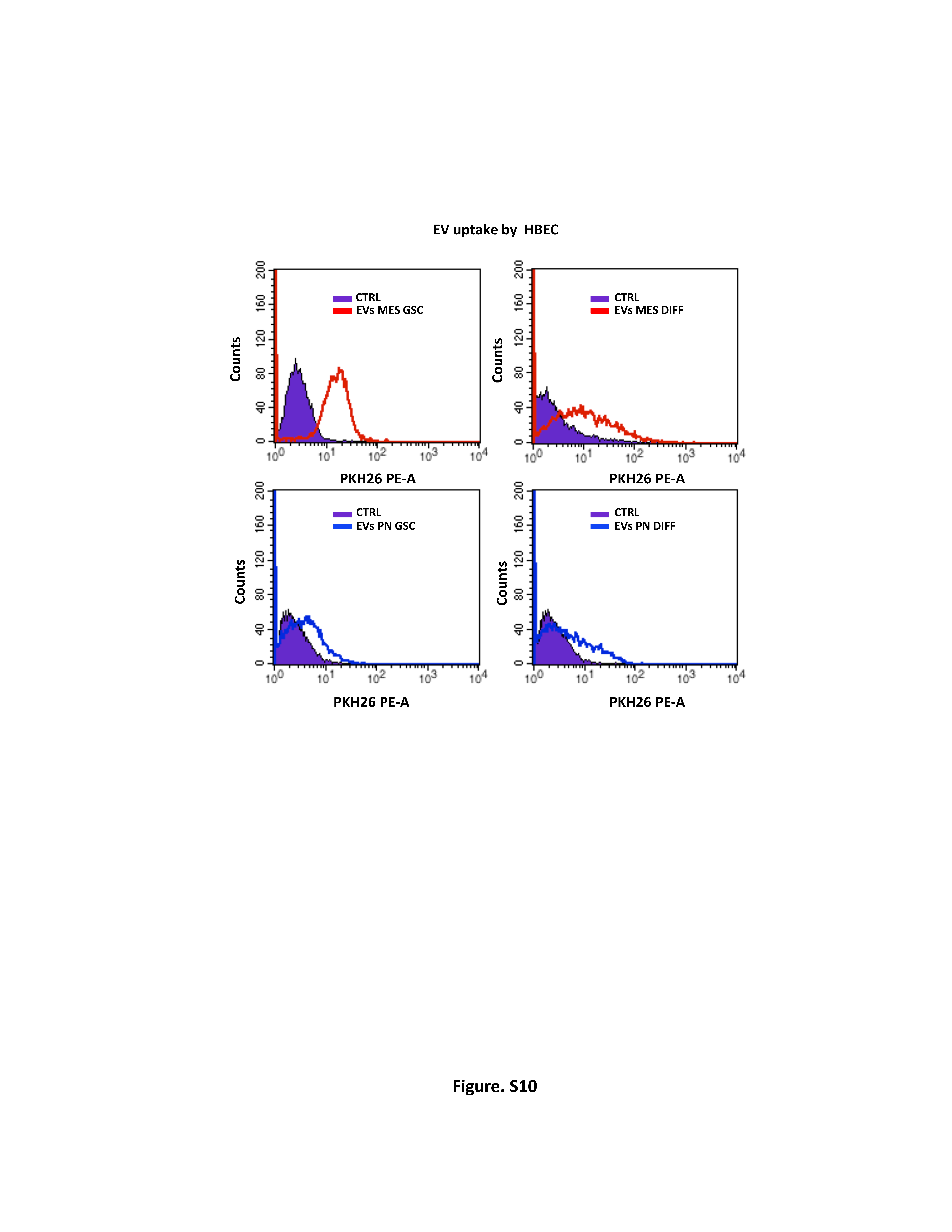

Supplement: Supplemental Material [file ZJEV_A_1490144_SM0503.zip › 0Spinelli_JEV_FINAL_new_graphs_jr_Page_22.tiff]
